# Neurotransmitter phenotype switching by spinal excitatory interneurons regulates locomotor recovery after spinal cord injury

**DOI:** 10.1038/s41593-022-01067-9

**Published:** 2022-05-06

**Authors:** Hannah Bertels, Guillem Vicente-Ortiz, Khadija El Kanbi, Aya Takeoka

**Affiliations:** 1 VIB-Neuroelectronics Research Flanders (NERF), Leuven, Belgium; 2 KU Leuven, Department of Neuroscience and Leuven Brain Institute, Leuven, Belgium; 3 imec, Leuven, Belgium

**Keywords:** Spinal cord injury, Spinal cord

## Abstract

Severe spinal cord injury in adults leads to irreversible paralysis below the lesion. However, adult rodents that received a complete thoracic lesion just after birth demonstrate proficient hindlimb locomotion without input from the brain. How the spinal cord achieves such striking plasticity remains unknown. In this study, we found that adult spinal cord injury prompts neurotransmitter switching of spatially defined excitatory interneurons to an inhibitory phenotype, promoting inhibition at synapses contacting motor neurons. In contrast, neonatal spinal cord injury maintains the excitatory phenotype of glutamatergic interneurons and causes synaptic sprouting to facilitate excitation. Furthermore, genetic manipulation to mimic the inhibitory phenotype observed in excitatory interneurons after adult spinal cord injury abrogates autonomous locomotor functionality in neonatally injured mice. In comparison, attenuating this inhibitory phenotype improves locomotor capacity after adult injury. Together, these data demonstrate that neurotransmitter phenotype of defined excitatory interneurons steers locomotor recovery after spinal cord injury.

## Abstract (web summary)

Bertels et al. identify that spinal neurons switch neurotransmitter phenotype from excitation to inhibition after spinal cord injury. Manipulation of neurotransmitter phenotype reveals that maintaining excitatory phenotype is essential for locomotor recovery.

## Main

Severe spinal cord injury disrupts connections between the brain and the spinal cord, which leads to the inability of body movements controlled by the spinal cord below the lesion. Consequently, spinal circuits require pharmacological agents, electrical stimulation or perineal/tail pinching to generate movements in adult rodents^1–3^ and electrical stimulation in humans^4–6^ after a functionally complete spinal cord lesion. However, these are only temporary aids to enable movement generation and do not overcome the obstacle of regaining long-term mobility after injury.

Intriguingly, although complete spinal cord lesion leads to paralysis in adult rodents, the same injury given to neonatal rodents results in a strikingly proficient hindlimb locomotor ability in adulthood, without any descending axon regeneration across the lesion or any form of external stimulation^7–9^. This ability to ‘walk without the brain’ involves spinal-cord-autonomous functions associated with increased excitability of spinal circuits below the lesion^10,11^, but the underlying mechanisms remain elusive. In addition, how the same insults imposed at different ages lead to divergent locomotor recovery is unknown, even though insights on these processes likely play a pivotal role in developing interventions that enhance locomotor recovery after spinal cord injury in the adult.

Studies with pharmacological perturbation suggest that regulation of excitation–inhibition (EI) balance of circuits below the lesion is a critical mechanism that defines the motor capacity of spinal-cord-injured cats and rodents, regardless of the age of injury^12–14^. Along with an injury-induced change in intrinsic motor neuron (MN) properties^15,16^, the balance of synaptic input to MNs is one of the determinants of MN excitability. Although severe injury increases inhibitory synaptic input to MNs innervating limb muscles below the lesion, long-term locomotor training reverses the increased inhibitory drive on MNs derived from the local interneurons^17–19^. This activity-dependent reorganization of EI input ratio from spinal interneurons to MNs likely contributes to locomotor improvement after long-term training. Furthermore, neuronal activity can trigger long-lasting modification of gene expression profiles that alter neurocircuit functions in a cell-type-specific manner, including neurotransmitter (NT) phenotype switches^20–23^. However, little has been explored on whether such activity-dependent mechanisms steer functionality of the spinal cord after injury and how age of injury shapes circuit reorganization.

After a complete spinal cord injury, somatosensory feedback is the only external input to the spinal circuits below the lesion. Among somatosensory populations, proprioceptive afferents (PAs) directly contact MNs and multiple classes of spinal interneurons to transmit movement-matched information necessary for motor control^24–27^. PA activity is essential for spinal circuit assembly and function during development^28,29^ and locomotor recovery after an incomplete spinal cord injury^30^. Given the role of proprioception in circuit development, motor control and recovery, PAs are in a prime position to direct locomotor circuits to a functional network after spinal cord injury in the absence of the brain.

In this study, we found that subsets of excitatory, but not inhibitory, spinal interneurons mediate the age of injury-dependent synaptic connectivity profiles to MNs. NT phenotype specification regulates spatially defined subsets of excitatory interneurons to maintain glutamatergic identity after neonatal injury and to gain inhibitory phenotype after adult injury. Furthermore, disrupting their NT identity from excitatory to inhibitory after neonatal injury abolishes the ability to walk without the brain, demonstrating that preserving the glutamatergic phenotype is essential. Using an intersectional genetic model, we found that removal of PAs disrupts glutamatergic phenotype of the subsets of excitatory interneurons after neonatal injury, indicating that continued glutamatergic phenotype expression is dependent on PA activity. Lastly, genetically attenuating inhibitory phenotype after an adult injury significantly improved defined locomotor parameters, especially when combined with locomotor training. Together, our study reveals age of injury-dependent flexible NT phenotype expression of specific classes of excitatory interneurons as critical neuronal substrates that steer locomotor capacity after severe spinal cord injury.

## Results

### Adult mice with neonatal injury exhibit proficient hindlimb locomotion

**Fig. 1:**
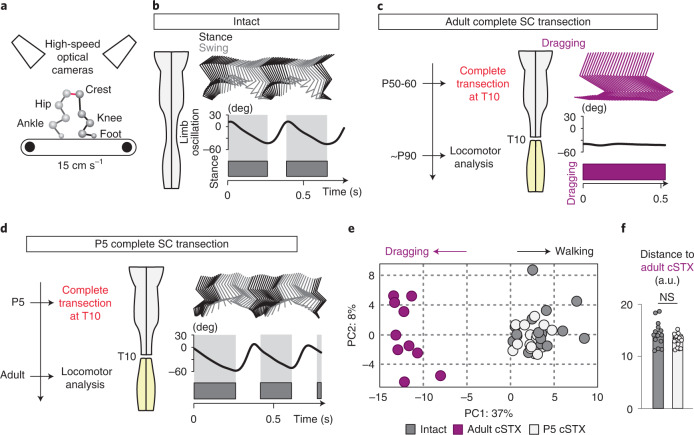
Adult mice with neonatal injury exhibit proficient locomotion. **a**, Kinematic recording setup with ten high-speed optical cameras monitoring the position of infrared reflective markers placed on hindlimb joints. **b**–**d**, Timeline, stick decomposition, limb oscillation and corresponding stance/swing phases of intact, adult cSTX and P5 cSTX mice. Dark gray horizontal bars indicate stance, and empty spaces correspond to swing. **e**, PC analysis for bipedal body-weight-supported treadmill locomotion shows that intact and P5 cSTX groups exhibit proficient locomotion, whereas adult cSTX mice do not (15 cm s^−1^, 15–25 gait cycles/timepoint/mouse; intact *n* = 15, adult cSTX *n* = 10 and P5 cSTX *n* = 18). Each dot represents one mouse. **f**, Histogram plot shows the average distance of each mouse from intact and P5 cSTX groups to the center of distribution of the adult cSTX group in the PC space (*P* > 0.05, two-sided unpaired *t*-test; intact *n* = 15 and P5 cSTX *n* = 18). Each dot represents one mouse. Error bars, s.e.m. a.u., arbitrary unit; deg, degree; NS, not significant; SC, spinal cord.

To determine the age of injury-dependent locomotor capacity, we subjected wild-type mice to a complete spinal cord transection (cSTX) at low thoracic level (~T10) either at postnatal day 5 (P5) or as an adult (~P60). We used ten high-speed cameras to quantitatively characterize hindlimb kinematics of each group together with intact controls during bipedal treadmill locomotion, all at 3–4 months of age (Fig. 1a–d). Although intact mice generated stereotypical alternating locomotion, mice that received cSTX as an adult (adult cSTX) exhibited complete and permanent paralysis of hindlimbs, examined up to 5 months after injury (Fig. 1b,c). In contrast, adult mice that received cSTX at P5 (P5 cSTX) displayed proficient hindlimb stepping without any externally applied stimulation (Fig. 1d and Supplementary Video 1).

**Extended Data Fig. 1:**
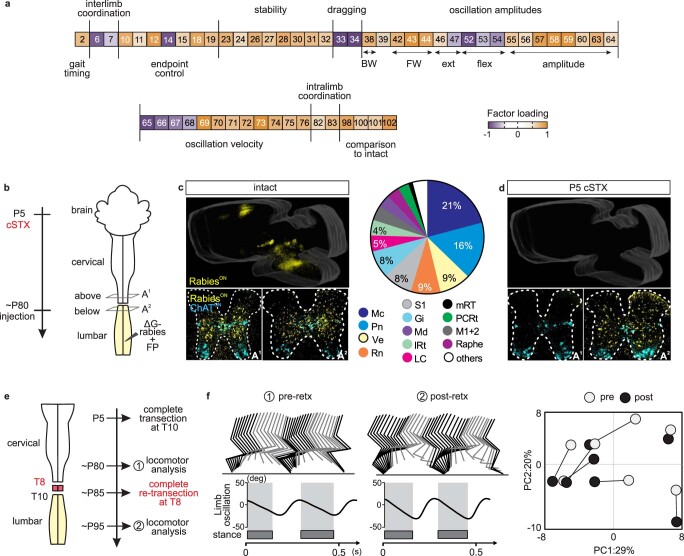
Mice with a neonatal injury learn to walk without the brain. **a**. Kinematic parameters (|factor loading|>0.5) that highly correlate to the locomotor capacity specific PC1 capture differentiation of dragging and walking. **b**. Scheme of retrograde tracing using genetically modified rabies tagged with a fluorescent protein (FP) to detect neuronal somata of projection neurons innervating lumbar segments in intact and P5 cSTX mice. White: co-localization of ChAT^ON^ and Rabies-FP^ON^. **c, d**. Representative coronal sections of mid-thoracic (T6–7; A^1^) and low thoracic (T12–13; A^2^) spinal segments and the brain from intact **(c)** and P5 cSTX **(d)** mice (n=3 each). Pie chart shows composition of lumbar projecting neurons from intact mice (in decreasing order); Mc (magnocellular reticular nucleus including LPGi, GiV, GiA and DPGi; lateral-para, ventral, alpha, dorsal-para parts), Pn (pontine reticular nucleus, including PnO, PnC and PnV; oral, caudal and ventral parts), Ve (vestibular nucleus including LVe, SpVe, MVe; lateral, spinal and medial parts), Rn (red nucleus including PR, PaR, RMC, RPC, RRF; pre-rubral field, pararubral, magnocellular, parvicellular parts and retrorubral field), S1 (the primary somatosensory cortex), Gi (gigantocellular reticular nucleus), LC (Locus coeruleus), IRt (intermediate reticular nucleus), M1+2 (the primary and secondary motor cortices, 4.3%), Md (medullary reticular nucleus including both ventral and dorsal parts, 3.6%), Raphe (3.5%), PCRt (parvocellular reticular nucleus, 3.1%), mRT (mescencephalic reticular formation, 1.3%) and others (any structures that consist smaller fraction than 1% of total Rabies^ON^ neurons). Scale bar: 100 µm. **e**. Timeline and scheme of the first complete transection, the initial kinematic recordings, retransection, and post-retransection kinematic recordings. **f**. Representative locomotor kinematics and PC analysis of P5 cSTX mice (n=7) at pre- (open circle) and post-retransection (dark circle) timepoints. Representative stick decomposition of hindlimb movement during stepping on a treadmill (15 cm/s). Below, limb oscillation and swing/stance timings are shown. Dark gray bars indicate stance, while empty spaces correspond to swing. Lines connecting a set of open and dark circles in the PC space represent one mouse. Retx: retransection.

We assessed hindlimb kinematics by high-resolution quantitative locomotor analyses^30^. We computed 102 parameters (Supplementary Table 1) and applied principal component (PC) analysis to all measured parameters/gait cycle/mouse to identify variability related to differences among intact, adult cSTX and P5 cSTX groups. The largest source of variance (PC1; 37%) was expectedly the difference between hindlimb dragging and stepping (Fig. 1e; that is, adult cSTX mice contrasting intact and P5 cSTX groups), with parameters related to persistent hindlimb dragging showing high correlation to PC1 (Extended Data Fig. 1a). In addition, the PC distance between intact or P5 cSTX groups to the adult cSTX group did not differ significantly (Fig. 1f). Therefore, we conclude that mice with neonatal injury walk without the brain with proficiency mostly indistinguishable from intact mice.

Locomotor proficiency detected in P5 cSTX mice is not due to establishing connections from the brain via axon regeneration across the complete lesion. We retrogradely labeled descending neurons projecting to the lumbar spinal cord in intact and adult mice with neonatal injury (Extended Data Fig. 1b). In intact mice, injection of G-deleted rabies to the lumbar segments labeled descending projection neurons originating from the brain and spinal cord (Extended Data Fig. 1c). In contrast, we did not detect rabies^ON^ neurons either in the spinal cord above the lesion or in the brain of P5 cSTX mice in adulthood, despite no obvious difference in neuronal infection efficiency within the local circuits between the two groups (Extended Data Fig. 1d). Furthermore, complete re-transection of the spinal cord a few segments rostral to the original lesion did not deteriorate locomotor patterns (Extended Data Fig. 1e,f and Supplementary Video 2). PC analysis applied to kinematic parameters recorded at before and after re-transection revealed that the largest source of variability was an inter-subject variability and not associated with the re-transection (Extended Data Fig. 1f). Therefore, the locomotor ability of adult mice with neonatal injury is attributed to the functionality of spinal circuits completely isolated from the brain.

### vGlut2^ON^ neurons undergo NT phenotype switch after adult injury

**Fig. 2:**
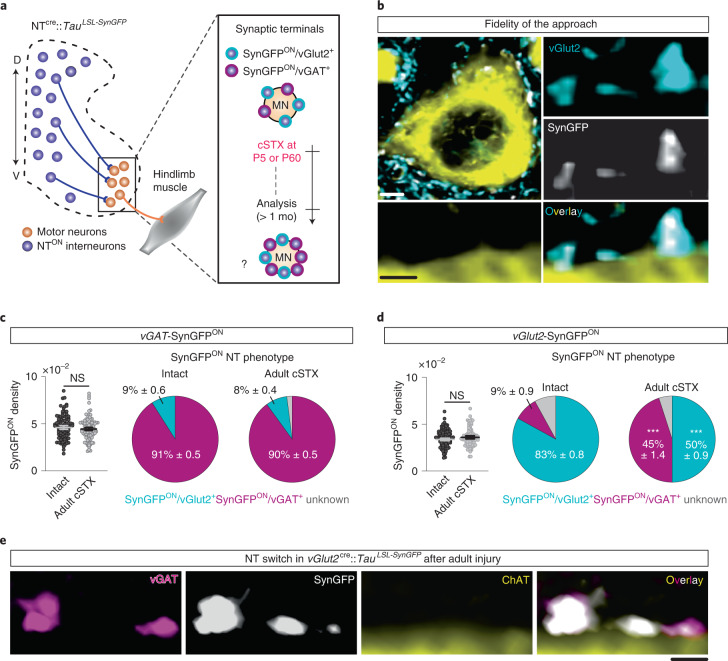
vGlut2^ON^ interneurons undergo NT phenotype switch after adult injury. **a**, Experimental scheme to visualize and quantify synaptic terminals derived from specific interneurons to MNs that innervate hindlimb muscles. **b**, Representative image of synaptic apposition of SynGFP^ON^ terminals to a ChAT^ON^ MN and co-localization of vGlut2 antibody in intact *vGlut2*^cre^::*Tau*^*LSL-SynGFP*^ spinal cord showing fidelity of the approach (0.2 µm optical section). Scale bars: top left, 5 µm; bottom left, 2 µm. **c**, **d**, Synaptic terminals derived from vGAT^ON^ (**c**) or vGlut2^ON^ (**d**) neurons depicted in the density of SynGFP^ON^ synaptic boutons (per µm^2^) apposing MNs (left) and % antibody labeling of NT expression (vGlut2^+^ or vGAT^+^) with SynGFP^ON^ genetic markings to visualize NT phenotype (right) of intact and adult cSTX spinal cords. Each dot represents one MN (>10 reconstructed MNs per NT analysis with antibody, per mouse, per genotype, *n* = 5 for each group, *P* > 0.05 for both *vGlut2*^cre^ and *vGAT*^cre^::*Tau*^*LSL-SynGFP*^ intercrosses, two-sided unpaired *t*-test). SynGFP^ON^ density is normalized to the surface area of measured ChAT^ON^ MNs. A low level of NT phenotype switch is detected in intact preparation for both intercrosses. Mean density of SynGFP^ON^/vGAT^+^ from each MN in *vGlut2*^*cre*^::*Tau*^LSL*-SynGFP*^ mice: intact 3.0 × 10^–3^ ± 0.7 terminals per μm^2^; adult cSTX 15.8 × 10^–3^ ± 0.4 terminals per μm^2^ (*P* < 0.001, two-sided unpaired *t*-test, and *P* < 0.001 for percentages of intact versus adult cSTX in *vGlut2*^*cre*^::*Tau*^LSL*-SynGFP*^). **e**, Genetically marked SynGFP^ON^ terminals co-immunolabeled for vGAT protein (vGAT^+^) in *vGlut2*^cre^::*Tau*^*LSL-SynGFP*^ spinal cord after adult injury. Error bars, s.e.m. Scale bar, 3 µm. D, dorsal; mo, month; NS, not significant; V, ventral.

Synaptic input from spinal interneurons to MNs is one of the factors regulating the final central nervous system (CNS) output that controls muscle contractions. As inhibitory synaptic input to MNs is known to increase after injury, we asked whether the EI synaptic input ratio from spinal interneurons to MNs that innervate hindlimb muscles depends on injury age. To visualize excitatory and inhibitory synaptic terminals, we crossed major excitatory or inhibitory NT Cre-driver lines (*vGlut2*^*cre*^ or *vGAT*^*cre*^) with *Tau*^*LSL-nlsLacZ-SynGFP*^ reporter mice to conditionally express synaptophysin tagged with green fluorescent protein (GFP) (SynGFP^ON^) in either excitatory or inhibitory spinal interneurons. This approach indelibly labeled synaptic terminals derived from either genetically glutamatergic vGlut2^ON^ or inhibitory vGAT^ON^ interneuron populations (Fig. 2a). This method shows high fidelity between genetically labeled excitatory or inhibitory terminals to assess protein expression of vGlut2 or vGAT with immuno-labeling (designated as vGlut2^+^ or vGAT^+^, as opposed to genetically labeled vGlut2^ON^ or vGAT^ON^) at the junction of choline acetyltransferase^ON^ (ChAT^ON^) MNs in intact spinal cords (>80%; Fig. 2b–d).

**Extended Data Fig. 2:**
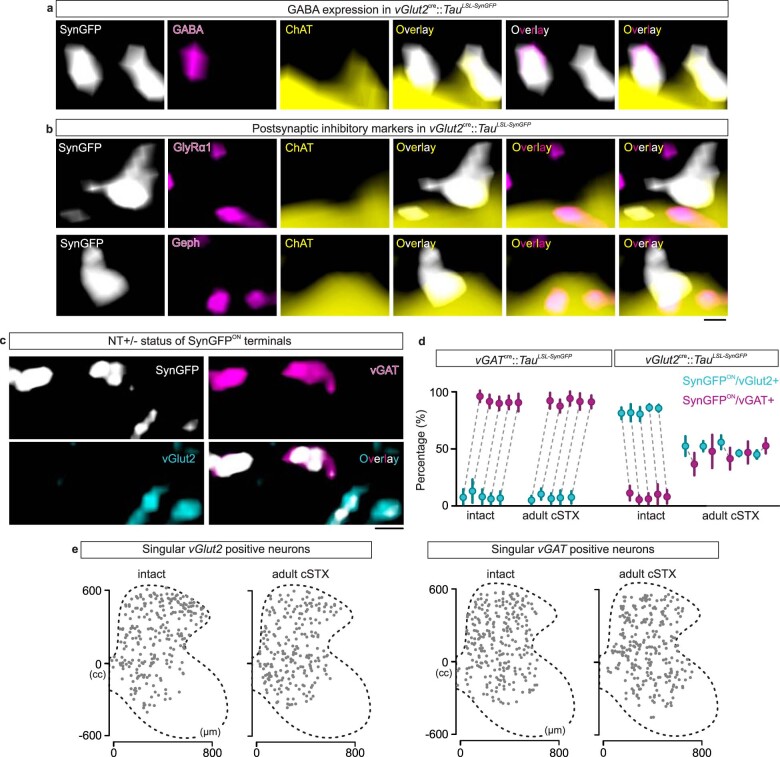
Neurotransmitter phenotype rearrangements after adult injury. **a, b**. Representative high-resolution images of (a) GABA expression in the genetically marked *vGlut2*-SynGFP^ON^ terminals and (b) postsynaptic inhibitory markers glycine receptor and gephyrin expression by ChAT^ON^ motor neurons. Post-synaptic markers are adjacent to SynGFP^ON^ terminals derived from genetically marked vGlut2^ON^ neurons in *vGlut2*^cre^::*Tau*^*LSL-SynGFP*^ spinal cord after adult injury (0.2 µm optical section). Scale bar: 2 µm. **c**. Triple immunolabeling of SynGFP^ON^, vGlut2^+^, and vGAT^+^ terminals in the adult cSTX spinal cord. NT: neurotransmitter. Scale bar: 2 µm. **d**. Quantification of average % SynGFP^ON^/vGlut2^+^ and SynGFP^ON^/vGAT^+^ to MNs of single mice in intact and adult cSTX (n=5 for each group for each intercross). Regardless of genetic markings or injury, vGlut2 and vGAT protein expression (vGlut2^+^ or vGAT^+^) at the synaptic terminals are mutually exclusive. Each mouse is represented with one vGlut2 and one vGAT dot with a connector. **e**. Location of neurons with singular *vGlut2* and *vGAT* expression for intact and adult cSTX experimental groups (1x16 µm sections). Error bars: ± 2SD.

Surprisingly, we detected no increase in the number of synaptic terminals derived from genetically marked inhibitory terminals in *vGAT*^*cre*^::*Tau*^LSL*-SynGFP*^ spinal cords after adult injury (Fig. 2c). Instead, we detected a five-fold increase of genetically marked excitatory terminals with vGAT^+^ expression (% SynGFP^ON^/vGAT^+^) in *vGlut2*^*cre*^::*Tau*^LSL*-SynGFP*^ spinal cords where almost half of the synaptic terminals derived from vGlut2^ON^ neurons became vGAT^+^ (Fig. 2d,e). This change accompanied the expression of GABA in terminals and post-synaptic inhibitory markers by MN, adjacent to apposition sites (Extended Data Fig. 2a,b). In parallel, we found a decrease in % of SynGFP^ON^/vGlut2^+^ while the total number of genetically marked SynGFP^ON^ terminals remained unchanged (Fig. 2d). Supporting this observation, we also found that vGlut2^+^ or vGAT^+^ expression at SynGFP^ON^ terminals is mutually exclusive (Extended Data Fig. 2c,d).

**Fig. 3:**
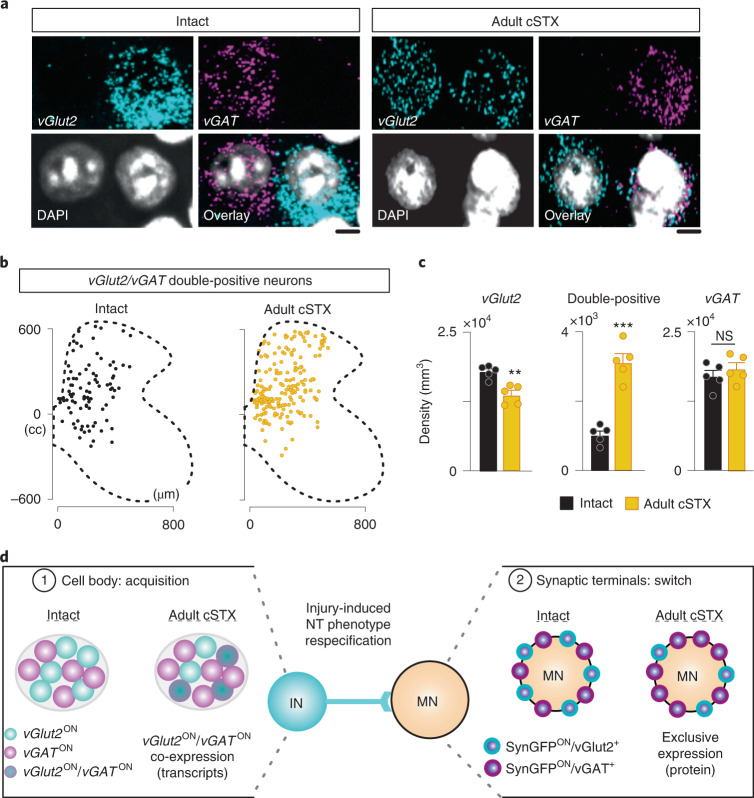
Dorsal and intermediate vGlut2^ON^ interneurons acquire vGAT phenotype after adult injury. **a**, Representative in situ hybridization example of the lumbar spinal cord (L4–6) sections with a singular expression of *vGlut2*, *vGAT* or double-positive (*vGlut2*/*vGAT*), marked with DAPI. Scale bar, 5 µm. **b**, Location of double-positive neurons with *vGlut2*/*vGAT* expression for intact and adult cSTX experimental groups (6 × 16-µm sections). **c**, Density of singular *vGlut2* and double-positive and singular *vGAT* neurons of intact and adult cSTX mice, normalized to gray matter volume (both groups *n* = 5, 2–3 sections per spinal cord). Each dot represents the mean of individual animals (**P < 0.01, ***P < 0.001, two-sided unpaired *t*-test). Mean percentages: intact: *vGlut2* 48%, double-positive 3%, *vGAT* 49%; adult cSTX: *vGlut2* 41%, double-positive 9%, *vGAT* 50%. **d**, Summary scheme represents co-expression of *vGlut2*/*vGAT* transcripts at the cell body and mutually exclusive expression of the two NTs at the synaptic terminals. IN, interneuron. Error bars, s.e.m.

A phenomenon of activity-dependent NT phenotype switch in the brain was reported previously^22,31^. To determine whether this observation at synaptic terminals is the case and, if so, whether the NT phenotype switch occurs uniformly among glutamatergic interneurons, we used in situ hybridization of *vGlut2* (*Slc17a6*) and *vGAT* (*Slc32a1*) mRNA. In an intact spinal cord, we detected neurons with a singular expression of *vGlut2* or *vGAT* transcripts in equal abundance with only a minor proportion of neurons co-expressing *vGlut2* and *vGAT* transcripts, distributed sparsely along the dorsoventral axis (Fig. 3a–c and Extended Data Fig. 2e). Together with the protein level analysis where we detected a low level of the opposite NT expression in genetically marked terminals (Fig. 2c,d), this result demonstrates that interneurons exhibit flexible NT phenotype in an intact spinal cord. Adult cSTX significantly increased the density of neurons with *vGlut2*/*vGAT* co-expression in the dorsal and intermediate lamina (Fig. 3b,c). This shift paralleled a decrease in neurons with a singular expression of *vGlut2* but no change in *vGAT* (Fig. 3c), a finding that suggests that a fraction of dorsal and intermediate excitatory interneurons acquires *vGAT* phenotype at the transcript level after adult injury.

Although co-expression of two distinct NT types by spinal MNs has been reported^32,33^, NT phenotype switch is a previously unknown mechanism in the mammalian spinal cord. Notably, NT phenotype switch not only refers to a complete replacement of one NT by another but can also refer to dynamic changes in the level of two or more NTs at the transcript and protein levels within a single neuron^23^. In addition, neurons can synthesize two or more NTs and release each NT selectively from different pre-synaptic terminals^34,35^. This is consistent with our observation, in which *vGlut2* and *vGAT* transcripts are co-expressed at the cell body, whereas protein accumulation at synaptic terminals is exclusive to either vGlut2 or vGAT (Fig. 3d). These findings demonstrate that the previously described increase in inhibitory input to MNs after adult injury^17,18^ is not due to sprouting of synapses derived from inhibitory interneurons (Fig. 2c) but, rather, to a NT phenotype switch of excitatory interneurons (Fig. 2d). Together, our results identified cell-type-specific NT phenotype switch as a pathophysiological response to a severe spinal cord injury affecting the mature nervous system.

### vGlut2^ON^ neurons undergo synaptic sprouting after neonatal injury

**Fig. 4:**
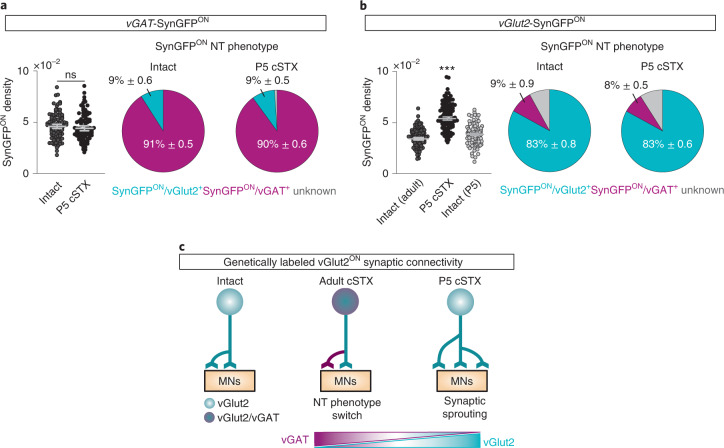
vGlut2^ON^ interneurons undergo synaptic sprouting after neonatal injury. **a**, **b**, Synaptic terminals derived from vGAT^ON^ (**a**) and vGlut2^ON^ (**b**) neurons depicted in density of SynGFP^ON^ synaptic boutons (per µm^2^) apposing MNs (left) and % antibody labeling of NT expression with SynGFP^ON^ genetic markings to visualize NT phenotype (right). Each dot represents one MN (>10 reconstructed MNs per NT analysis with antibody, per mouse, *n* = 5 for each group). SynGFP^ON^ density is normalized to the surface area of measured ChAT^ON^ MNs. Intact data are identical to Fig. 2c,d. **a**, *P* > 0.05, two-sided unpaired *t*-test. **b**, Mean of density computed from each MN: SynGFP^ON^/vGlut2^+^: intact 3.2 × 10^–2^ ± 0.5 terminals per μm^2^, P5 cSTX 4.6 × 10^–2^ ± 0.2 terminals per μm^2^, P5 intact 3.3 × 10^–2^ ± 0.2 terminals per μm^2^ (*P* < 0.001, two-sided unpaired *t*-test); SynGFP^ON^/vGAT^+^: intact 3.0 × 10^–3^ ± 0.2 terminals per μm^2^, P5 cSTX 4.5 × 10^–3^ ± 0.4 terminals per μm^2^, P5 intact 2.4 × 10^–3^ ± 0.1 terminals per μm^2^ (*P* > 0.05, one-way ANOVA with Tukey’s post hoc test). **c**, Summary diagram of NT phenotype switch and synaptic sprouting of genetically labeled vGlut2^ON^ interneurons. Error bars, s.e.m.

Next, we determined whether a similar NT phenotype switch occurred after neonatal injury. Much in contrast, we found no evidence of NT phenotype switch after neonatal injury. Instead, we observed a significant increase in genetically marked glutamatergic SynGFP^ON^ terminals to MNs in *vGlut2*^*cre*^::*Tau*^*LSL-SynGFP*^ mice (Fig. 4a,b). This increase is due to synaptic sprouting in response to injury but not injury-induced disruption of synaptic pruning that often occurs during circuit development^36^, because we found a significantly lower number of SynGFP^ON^ inputs to MNs in intact spinal cords at P5 than in spinal cords upon neonatal injury (Fig. 4b).

Together, the two injury models revealed that the age of injury defines two opposing synaptic connectivity profiles of excitatory interneurons to MNs. We found NT switch after adult injury, which led to high vGAT^+^ synaptic input to MNs in contrast to genetically marked glutamatergic synaptic sprouting after neonatal injury (Fig. 4c). These observations raise questions about cell type specificity among excitatory interneurons that participate in synaptic connectivity reorganizations, which circuit components direct these plasticity mechanisms and whether NT phenotype specification determines locomotor capacity after injury.

### vGlut2^ON^ synaptic reorganization to MNs is subpopulation specific

**Fig. 5:**
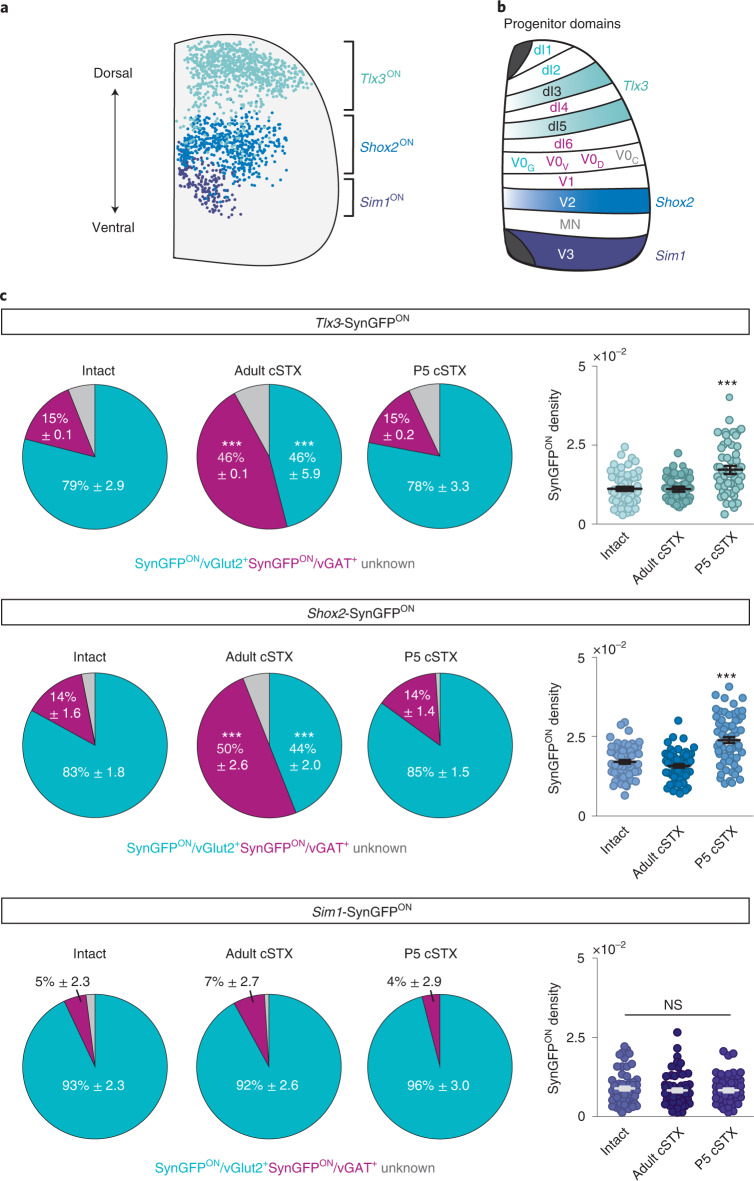
Age of injury-dependent vGlut2^ON^ synaptic profile is subpopulation specific. **a**, **b**, Scheme of analyzed excitatory subpopulations and their positions in the mature lumbar spinal cord (**a**) and transcription factor code of spinal cord PDs. PDs that give rise to vGlut2^ON^ interneurons are in magenta, vGAT^ON^ interneurons in cyan and cholinergic interneurons and MNs in gray (**b**, PD; dI1–dI6, V0–V3 and MN, examined excitatory subpopulations: light blue *Tlx3*^ON^, blue *Shox2*^ON^ and purple *Sim1*^ON^). **c**, Quantification of synaptic terminals derived from *Tlx3*^ON^, *Shox2*^ON^ and *Sim1*^ON^ neurons depicted in NT phenotype identity (left; vGlut2^+^) and density of SynGFP^ON^ synaptic boutons (per µm^2^) apposing MNs (right) in intact, adult cSTX and P5 cSTX spinal cords. Each dot represents one MN (>10 reconstructed MNs per NT analysis with antibody, per mouse, per genotype, *n* = 3 for each group). SynGFP^ON^ density is normalized to the surface area of measured ChAT^ON^ MNs (*P* < 0.001, one-way ANOVA with Tukey’s post hoc test). Error bars, s.e.m. NS, not significant.

*vGlut2* and *vGAT* transcript expression after adult injury reveals that specific vGlut2^ON^ neurons residing in the dorsal and intermediate lamina are driving the process of NT phenotype switch (Fig. 3b). To gain genetic access to different subpopulations of excitatory interneurons residing along distinct dorsoventral positions in the spinal cord (Fig. 5a), we used stratification by developmental origin^37^. We used three transgenic mouse lines, each expressing Cre-recombinase under the control of a different progenitor domain (PD)-specific transcription factor (PD^cre^; Fig. 5b) and intercrossed with the *Tau*^LSL-*SynGFP*^ reporter line to selectively visualize synaptic output derived from the selected PD population.

**Extended Data Fig. 3:**
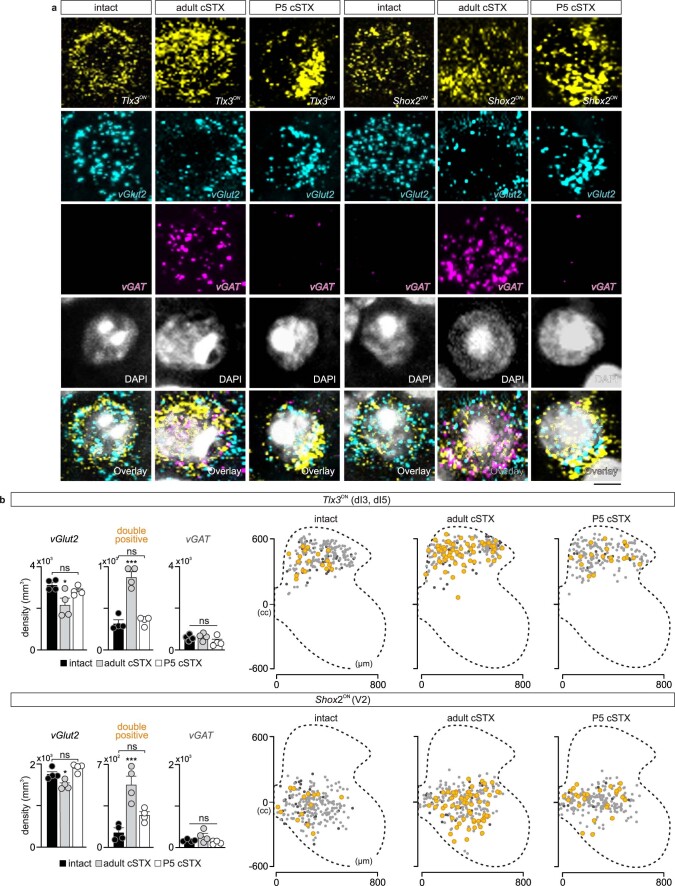
Cell type-specific NT profiles of genetically marked vGlut2^ON^ interneurons. **a**. Example of multiplex in situ hybridization of lumbar spinal cord sections from PD^cre^::*Rosa*^*LSL*-TdTomato^ mice. Intact, adult cSTX and P5 cSTX spinal cords with representative cells with PD^ON^/*vGlut2*^ON^ and PD^ON^/*vGlut2*^ON^/*vGAT*^ON^ expression, marked with DAPI. Scale bar: 5 µm. **b**. Left: density histograms of three different categories of PD^ON^ cells (left); singular *vGlut2*, double positive and singular *vGAT* normalized to gray matter volume (n=4 for each condition for each PD domain neurons, 2–3 sections per spinal cord). Each dot represents the mean of individual animals. Right: spatial distribution (*Tlx3*^ON^ 3×16 µm and *Shox2*^ON^ 5×16 µm thick section; light grey: PD^ON^*/vGlut2*^ON^, dark grey: PD^ON^*/vGAT*^ON^ yellow: PD^ON^*/vGlut2*^ON^*/vGAT*^ON^, *p<0.05, ***p<0.001, one-way ANOVA with Tukey’s post-hoc analysis). Mean percentages for *Tlx3*^ON^ population; intact: *vGlut2* 76%, double-positive 10%, *vGAT* 14%; adult cSTX: *vGlut2* 59%, double-positive 24%, *vGAT* 17%; P5 cSTX: *vGlut2* 80%, double-positive 10%, *vGAT* 10%. Mean percentages for *Shox2*^ON^ population; intact: *vGlut2* 82%, double-positive 7%, *vGAT* 11%; adult cSTX: *vGlut2* 66%, double-positive 23%, *vGAT* 11%; P5 cSTX: *vGlut2* 84%, double positive 10%, *vGAT* 6%. Error bars: SEM.

We found that both PD identity and age of injury determined the NT profile of their synaptic output. After the adult injury, *Tlx3*^ON^ (dI3 and dI5) and *Shox2*^ON^ (V2a), but not *Sim1*^ON^ (V3), interneurons exhibited increased SynGFP^ON^/vGAT^+^ and decreased SynGFP^ON^/vGlut2^+^ terminals to MNs (Fig. 5c). In parallel, we performed gene expression analysis using multiplex in situ hybridization of *vGlut2* and *vGAT* mRNA specifically on genetically labeled *Tlx3* or *Shox2* neurons (Extended Data Fig. 3a,b). In intact spinal cords, we detected that most *Tlx3*^ON^ or *Shox2*^ON^ neurons displayed singular expression of *vGlut2* transcripts, with a minor proportion of neurons with a singular expression of *vGAT* transcripts or co-expression of *vGlut2* and *vGAT* transcripts. In contrast, we found a significantly higher density of *vGlut2*/*vGAT* double-positive neurons in both *Tlx3*^ON^ and *Shox2*^ON^ neurons in adult cSTX spinal cords. This change was accompanied by a reduction in neurons with a singular expression of *vGlut2* transcripts and no change in the number of neurons with a singular expression of *vGAT* transcripts. Consistent with the protein level findings, we conclude that a fraction of *Tlx3*^ON^ or *Shox2*^ON^ excitatory interneurons acquires an inhibitory NT phenotype after adult injury.

After neonatal injury, all three PD populations maintained their glutamatergic phenotype at protein and transcript levels, consistent with the analysis on pan-vGlut2^ON^ interneurons (Fig. 4b and Extended Data Fig. 3a,b). Interestingly, we found that the neonatal injury-dependent synaptic sprouting was also subpopulation specific (Fig. 5c). We detected more SynGFP^ON^ boutons from *Tlx3*^ON^ and *Shox2*^ON^, but not *Sim1*^ON^, interneurons to MNs in P5 cSTX compared to the intact spinal cord. Together, the age of injury-specific NT identity switches and connectivity rearrangements to MNs occur with remarkable specificity among excitatory interneurons.

### PA ablation disrupts *vGlut2* identity after neonatal injury

**Fig. 6:**
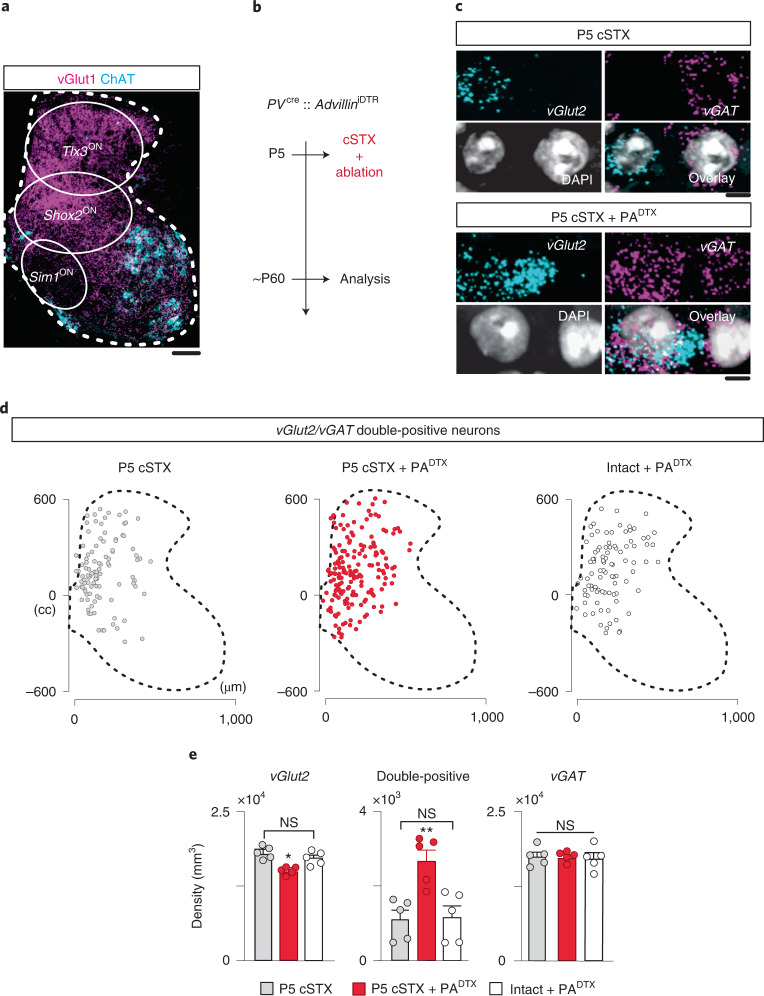
PA ablation disrupts *vGlut2* identity after neonatal injury. **a**, Spatial positioning of *Tlx3*^ON^, *Shox2*^ON^ and *Sim1*^ON^ interneurons in relation to vGlut1^ON^ afferent projection patterns. vGlut1 marks a mixture of PAs and a minor subpopulation of cutaneous afferents in the dorsal horn and PAs in the intermediate and ventral lamina. Scale bar, 100 µm. **b**, Experimental timeline of P5 cSTX, PA ablation and analysis. **c**, Example of in situ hybridization of lumbar spinal cord (L4–6) sections. P5 cSTX and P5 cSTX+PA^DTX^ spinal cords with representative cells with a singular expression of *vGlut2* and *vGAT*, or double-positive (*vGlut2*/*vGAT*), marked with DAPI. Scale bar, 5 µm. **d**, Location of *vGlut2/vGAT* double-positive cells for P5 cSTX, P5 cSTX+PA^DTX^ and intact+PA^DTX^ experimental groups (6 × 16 µm). **e**, Density of singular *vGlut2* and double-positive and singular *vGAT* normalized to gray matter volume (*n* = 5 for each group, 2–3 sections per spinal cord). Each dot represents the mean of individual animals (*P < 0.05, **P < 0.01, one-way ANOVA with Tukey’s post hoc test). Mean percentages: P5 cSTX: *vGlut2* 49%, double-positive 3%, *vGAT* 48%; P5 cSTX+PA^DTX^: *vGlut2* 42%, double-positive 8%, *vGAT* 50%; intact+PA^DTX^: *vGlut2* 48%, double-positive 3%, *vGAT* 59%. Error bars, s.e.m. NS, not significant.

**Extended Data Fig. 4:**
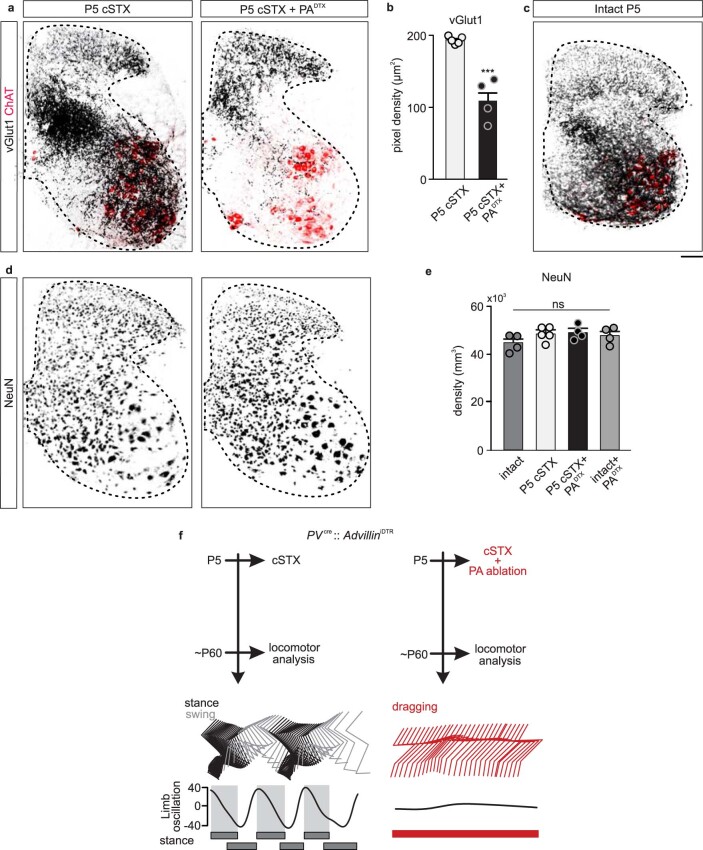
The approach to eliminate PAs. **a, b**. vGlut1 immunoreactivity and density quantification (P5 cSTX n=5, P5 cSTX+PA^DTX^ n=4). Each dot represents the mean of individual animals (p<0.001, two-sided unpaired t-test). In P5 cSTX+PA^DTX^ spinal cord, sparse unablated Ia afferents remain in the ventral horn. **c**. Representative image of vGlut1 innervation pattern at intact P5. **d, e**. NeuN immunoreactivity and density quantification (intact n=5, P5 cSTX n=5, P5 cSTX+PA^DTX^ n=4, intact+PA^DTX^ n=4). Each dot represents the mean of individual animals. (p>0.05, one-way ANOVA with Tukey’s post-hoc analysis). **f**. Timeline, stick decomposition, endpoint trajectory, limb oscillation, and corresponding stance/swing phases of adult *PV*^cre^*::Advillin*^*iDTR*^ mice that received cSTX at P5 with or without PA ablation. Error bars: SEM. Scale bar: 100 µm.

Excitatory interneurons that undergo plastic synaptic sprouting and NT phenotype switches reside primarily within the dorsal and intermediate, but not in the ventral, spinal cord. Under normal circumstances, these neurons receive high vesicular glutamate transporter 1 (vGlut1)^ON^ somatosensory input (Fig. 6a). Given the role of PAs in circuit development and motor recovery, we probed whether PAs might maintain vGlut2 phenotype after neonatal injury. We used an intersectional approach, an intercross of *Parvalbumin* (*PV*)^*cre*^ and *Advillin*^*iDTR*^ mouse lines, to express human diphtheria toxin (DTX) receptor (DTR) in PV^ON^/Advillin^ON^ somatosensory afferents^30^. This approach enabled ablation of PAs with DTX at the time of injury at P5 (P5 cSTX+PA^DTX^), which led to a substantial reduction of central PAs marked by vGlut1^ON^ terminals while leaving the minor non-proprioceptive somatosensory vGlut1^ON^ terminals unaffected (Fig. 6b and Extended Data Fig. 4a–c). In contrast to P5 cSTX mice, P5 cSTX+PA^DTX^ mice expectedly showed severe locomotor impairments, without any obvious unspecific neuronal degeneration within local circuits (Extended Data Fig. 4d–f).

To determine whether PA ablation leads to NT phenotype switch after neonatal injury, we reconstructed the spatial distribution of *vGlut2* and *vGAT* mRNA in P5 cSTX, P5 cSTX+PA^DTX^ and intact+PA ablation alone at P5 (intact+PA^DTX^) groups. Although P5 cSTX alone does not alter the transcript ratio, P5 cSTX combined with PA ablation showed a marked increase in neurons with *vGAT*/*vGlut2* co-expression in the dorsal and intermediate lamina, like the adult cSTX group (Fig. 6c–e). This shift paralleled a decrease in neurons with a singular expression of *vGlut2* but no change in a singular expression of *vGAT* (Fig. 6e). Because the EI transcript profile in intact+PA^DTX^ resembled that of intact and P5 cSTX alone groups (Fig. 6d,e), we conclude that combining the lesion and PA ablation shift EI gene expression profiles after neonatal injury.

Together, we found that PAs are necessary to sustain a singular expression of *vGlut2* by a subset of *vGlut2* neurons located mainly in the dorsal and intermediate lamina after neonatal injury. In contrast, the NT phenotype of ventral *vGlut2* neurons that receive less PA input appears to be independent of PA activity. The similarity in the spatial distribution of *vGAT*/*vGlut2* double-positive neurons detected in P5 cSTX+PA^DTX^ to those of adult cSTX spinal cords suggests that these double-positive neurons are most likely the same pool of interneurons that undergo a NT phenotype switch after adult injury.

### Overexpression of vGAT disrupts proficient locomotion after neonatal injury

**Fig. 7:**
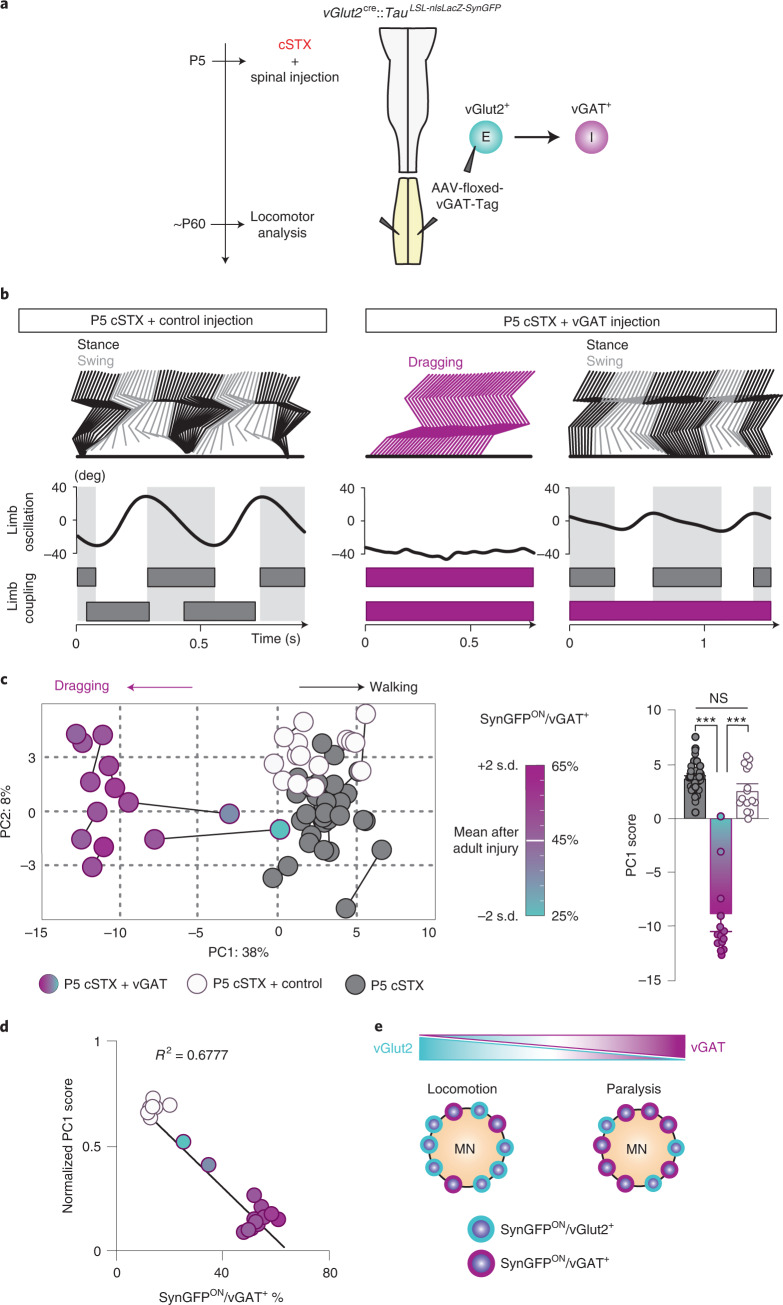
Overexpression of vGAT disrupts the ability to walk without the brain after neonatal injury. **a**, Timeline and experimental strategy to transduce *vGAT* expression in *vGlut2*^cre^ spinal interneurons in the lumbar spinal cord. **b**, Stick decomposition, limb oscillation and corresponding stance/swing phases of P5 cSTX+control injection and P5 cSTX+vGAT mice. Dark gray horizontal bars indicate stance, and empty spaces correspond to swing. Magenta bars indicate complete dragging of the hindlimbs. **c**, PC analysis was applied on mean values of 94 gait parameters (average of 6–15 steps per timepoint, *n* = 16 for P5 cSTX, *n* = 8 for P5 cSTX+control, *n* = 7 for P5 cSTX+vGAT). Each dot represents limb kinematics of either left or right hindlimb of one mouse. The % vGAT^+^ is normalized to the mean of % vGAT^+^ after adult injury (45%) and includes any limbs with SynGFP^ON^/vGAT^+^ % in the range of ±2 standard deviations. Color gradient ranges from 65% to 25%, representing the highest (magenta) and the lowest (cyan) % SynGFP^ON^/vGAT^+^. PC1 scores of P5 cSTX+vGAT showed a significant difference compared to P5 cSTX and P5 cSTX+control injection (***P < 0.001, one-way ANOVA with Tukey’s post hoc analysis). **d**, Linear regression analysis of locomotor capacity and NT composition of SynGFP^ON^ synaptic terminals in P5 cSTX mice with control or AAV-vGAT injections. Individual PC1 values were normalized using z-score and scaled to [0,1]. Statistical analysis showed a significant deviation of the slope from zero (*P* < 0.001). **e**, Summary diagram showing a linear correlation of NT phenotype and locomotor capacity. Error bars, s.e.m. deg, degree; NS, not significant.

**Extended Data Fig. 5:**
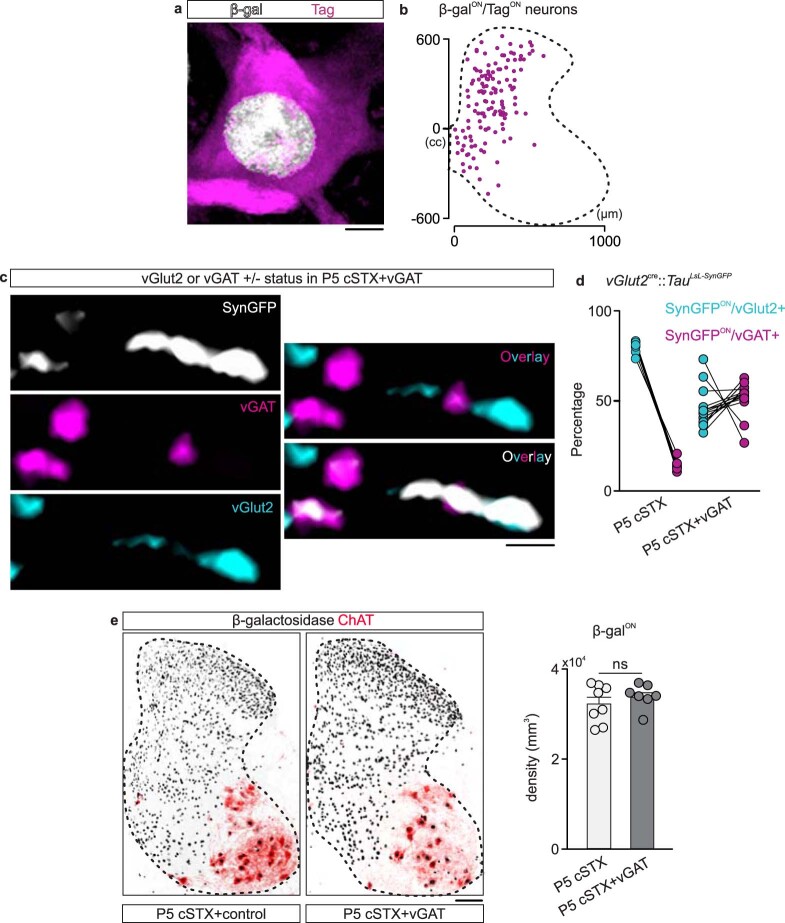
Virus-mediated expression of vGAT in genetically marked vGlut2^ON^ neurons and tissue integrity after intraspinal injections. **a**. Representative image showing a β-galactosidase^ON^ neuron with AAV-vGAT-Tag expression. Scale bar: 5 µm. **b**. Representative distribution of β-gal^ON^/Tag^ON^ cells in the P5+vGAT spinal cord. **c**. Representative confocal image of SynGFP^ON^, vGlut2^+^, and vGAT^+^ terminals with triple immunolabeling in P5 cSTX+vGAT spinal cord. Scale bar: 3 µm. **d**. Quantification of % SynGFP^ON^/NT^+^ in P5 cSTX versus P5 cSTX+vGAT groups (P5 cSTX n=7 4 mice per hemicord, P5 cSTX+vGAT n=7 all mice per hemicord). **e**. Representative image and quantification of β-galactosidase^ON^ nuclei distribution near the injection site in P5 cSTX+control and P5 cSTX+vGAT groups (P5 cSTX n=8, P5 cSTX+vGAT n=7). Each dot represents the mean of individual animals. (p>0.05, two-sided unpaired t-test). Error bars: SEM.

PA activity-dependent NT phenotype of spinal excitatory interneurons after neonatal injury raised a question of whether maintaining vGlut2^ON^ phenotype is essential for proficient stepping without the brain. To establish this link, we performed an intraspinal injection of LoxP-flanked AAVs in the lumbar spinal cord to express vGAT (AAV-DIO-vGAT-Tag) by excitatory neurons (Fig. 7a). As our vGlut2 subpopulation-specific analysis (Fig. 5) does not exhaustively examine other dorsal and intermediate vGlut2 subpopulations (that is, dI1, dI2 and V0_G_ PDs; Fig. 5b), we chose to use *vGlut2*^*cr*e^::*Tau*^*LSL-nlsLacZ-SynGFP*^ mice for this experiment while establishing inclusion criterion with 90%> infected vGlut2^ON^ neurons are restricted within dorsal and intermediate lamina. This approach allows us to simultaneously manipulate both *Tlx3*^cre^ and *Shox2*^cre^ populations as well as other dorsal and intermediate vGlut2^ON^ interneurons that may also contribute to recovery after neonatal injury (Fig. 5b). Broad bilateral injection of the virus across lumbar segments led to expression of the Tag at the cell body and mutually exclusive expression of vGAT^+^ and vGlut2^+^ at SynGFP^ON^ terminals (that is, a proportional increase in vGAT^+^ expression paralleled by a decrease in vGlut2^+^ expression; Extended Data Fig. 5a–e). We then examined the locomotor ability of P5 cSTX mice with AAV-DIO-vGAT-Tag (P5 cSTX+vGAT) or control injection of AAV-DIO-Tag (P5 cSTX+control) at ~P60 (Fig. 7a).

Virus-mediated expression of vGAT by dorsal and intermediate excitatory interneurons after neonatal injury deteriorated locomotor performance. Although P5 cSTX+control mice displayed alternation between left and right limbs, P5 cSTX+vGAT mice largely failed to develop a brain-independent locomotor capacity (Fig. 7b and Supplementary Video 3). However, we observed unilateral, non-weight-bearing reflexive steps on rare occasions (Fig. 7b, right). Therefore, we reconstructed SynGFP^ON^ terminals to MNs and quantified their NT phenotype as a proxy to validate viral transduction efficiency. We applied PC analysis on 94 parameters and then matched the % vGAT expression at synaptic terminals to MNs for each examined limb. Reconstructed PC space segregated experimental groups based on variability related to dragging-related parameters (Fig. 7c). Furthermore, limb kinematics during treadmill locomotion broadly matched the extent of viral efficiency among experimental mice—that is, hindlimbs with low vGAT^+^ and high vGlut2^+^ % synaptic inputs to MNs display ambulatory phenotype (Fig. 7d). Therefore, we conclude that acquiring vGAT accompanied by reduction of vGlut2 phenotype (Extended Data Fig. 5c,d) by dorsal and intermediate glutamatergic interneurons deteriorates locomotor ability after neonatal injury.

Together, our results demonstrate that vGAT expression by glutamatergic interneurons in the dorsal and intermediate lamina after injury contributes to paralysis, regardless of whether it is due to spontaneous shift after adult injury or virus-mediated shift after neonatal injury (Fig. 7e).

### Suppression of vGAT improves locomotor kinematics after adult injury

**Fig. 8:**
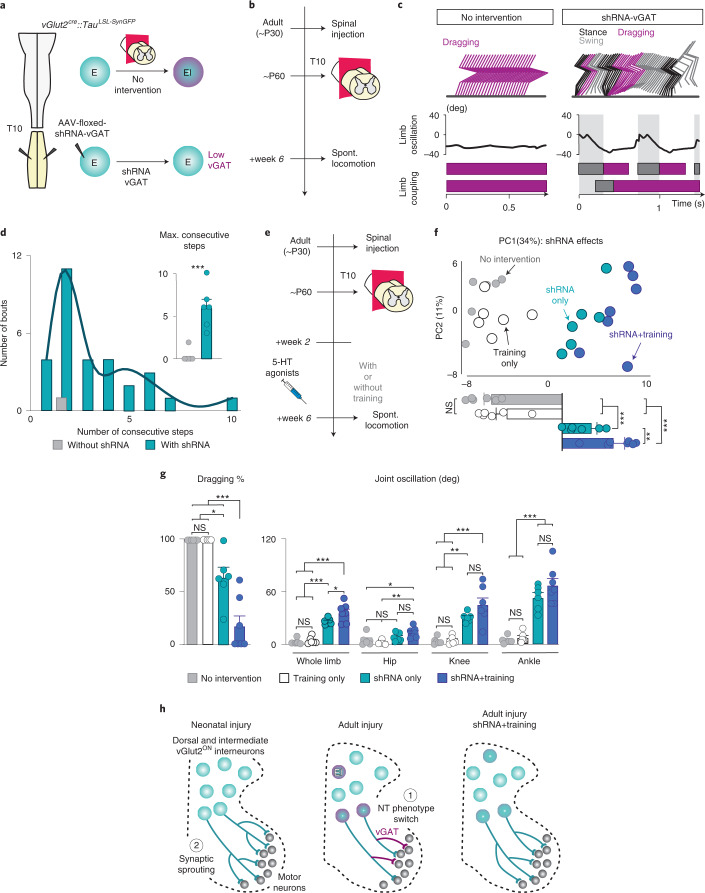
Suppression of vGAT expression by excitatory interneurons combined with locomotor training facilitates locomotor recovery after adult injury. **a**, **b**, Experimental strategy and timeline to attenuate *vGAT* expression in genetically labeled *vGlut2*^ON^ spinal interneurons after adult injury. **c**, Representative stick decomposition of adult cSTX mice with control (left) and shRNA-vGAT (right) injections at week 6. **d**, Frequency plot of stepping bouts and quantification of the maximum number of consecutive steps during a 30-second recording session (*n* = 6 for each group, *P* < 0.001 two-sided unpaired *t*-test). The line indicates a spline fit of the histogram distribution from the shRNA group. **e**, Timeline to induce stepping after adult injury with or without daily locomotor training. **f**, PC analysis of spontaneous stepping at week 6 (*n* = 6 each for no intervention, training only and shRNA-vGAT only and *n* = 7 for shRNA-vGAT+training). Each dot represents one mouse. Histogram plots below the PC reconstruction show differences in PC1 scores (*P < 0.05, ***P < 0.001 one-way ANOVA with Tukey’s post hoc analysis). **g**, Examples of kinematic parameters with additive effects of daily training combined with shRNA-vGAT (*n* = 6 each for no intervention, training only and shRNA-vGAT only and *n* = 7 for shRNA-vGAT+training; *P < 0.05, **P < 0.01, ***P < 0.001 one-way ANOVA with Tukey’s post hoc analysis). **h**, Age of injury-dependent circuit signatures capture decreasing vGlut2 input due to NT re-specification of dorsal and intermediate excitatory interneurons after adult injury (1) in contrast to increasing vGlut2 pre-motor synaptic input derived from the same population to MNs after neonatal injury (2). shRNA-vGAT and training interventions respectively minimize NT phenotype switch and facilitate synaptic sprouting, therefore likely contributing to added effects of recovery when combined. Error bars, s.e.m. deg, degree; NS, not significant; Spont., spontaneous.

**Extended Data Fig. 6:**
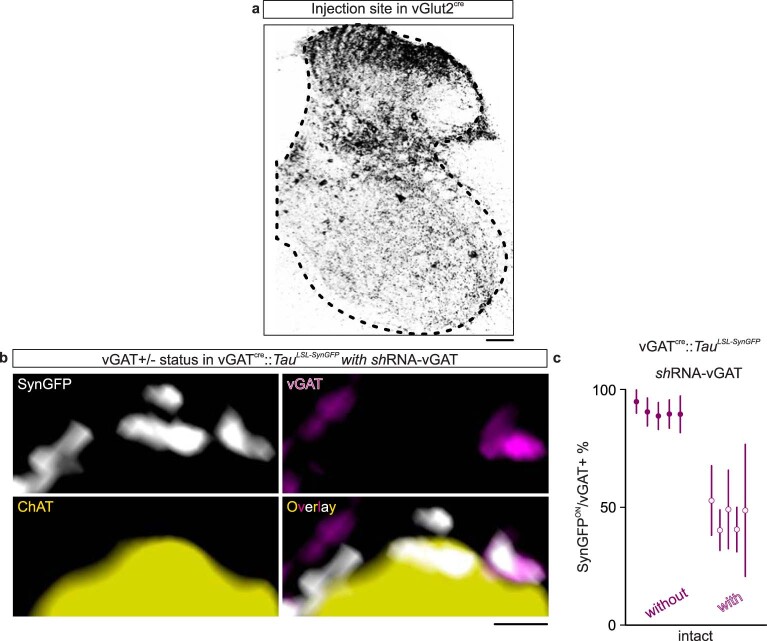
The approach to suppress vGAT gene expression after adult injury. **a**. Representative image of an injection site with GFP^ON^ neurons representing AAV-floxed-shRNA-vGAT-GFP infected vGlut2^ON^ neurons from *vGlut2*^cre^ mouse. Scale bar: 100 µm. **b**. Representative image of synaptic apposition of SynGFP^ON^ terminals to a ChAT^ON^ motor neuron and colocalization of vGAT antibody, or lack thereof, in intact vGAT^cre^::*Tau*^*LSL-SynGFP*^ mouse after shRNA-vGAT injection. Scale bar: 2 µm. **c**. Quantification of average % SynGFP^ON^/vGAT^+^ to MNs with or without the *sh*RNA-vGAT virus injection in intact *vGAT*^cre^::*Tau*^*LSL-SynGFP*^ mice (n=5 for each group, control 91 ± 0.52 %, *sh*RNA-vGAT 46 ± 2.5 %). Each data point represents 1 mouse, with the error bar indicating ± 2 SD of SynGFP^ON^/vGAT^+^ input density per MN (n=10 MN per mouse).

Having established that defined subpopulations of vGlut2 interneurons undergo an NT phenotype switch after adult injury and that the maintenance of vGlut2 NT phenotype is critical for proficient locomotion after neonatal injury, we asked whether attenuating vGAT^+^ expression facilitates locomotor improvement after adult injury. To conditionally downregulate vGAT expression in vGlut2 interneurons, we injected floxed AAVs encoding short-hairpin RNA (shRNA) against vGAT gene expression in the lumbar segments (shRNA-vGAT-Tag; Fig. 8a). This approach allows us to express shRNA-vGAT in Cre^ON^ neurons selectively (Extended Data Fig. 6a). We established the efficiency of this approach using intact *vGAT*^*cr*e^::*Tau*^*LSL -SynGFP*^ mice with genetic labeling of synaptic terminals derived from vGAT^ON^ interneurons combined with vGAT immunolabeling (vGAT^+^; Extended Data Fig. 6b,c).

**Extended Data Fig. 7:**
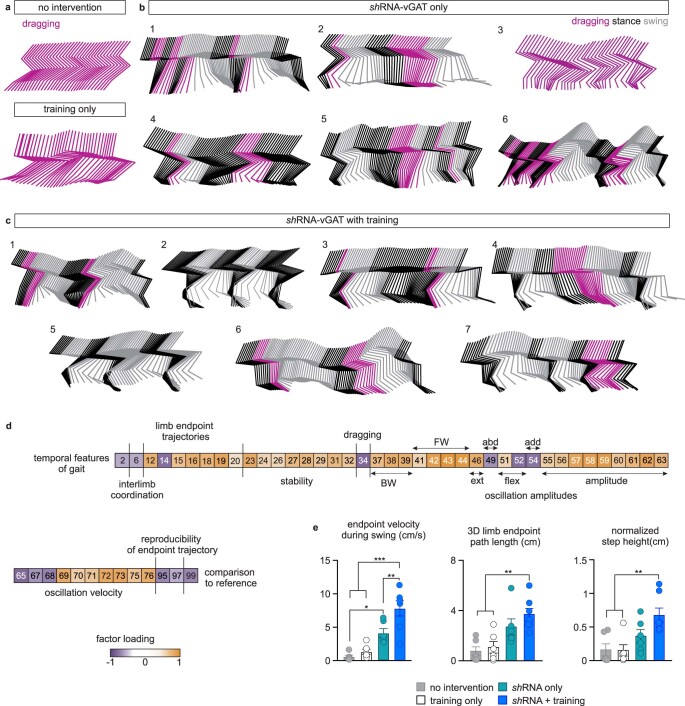
Additive facilitation of locomotor proficiency with suppression of *vGAT* gene expression and locomotor training after adult injury. Representative stick decomposition of adult cSTX mice without any intervention or training only at week 6. **b**, **c**. Stick decomposition of individual adult cSTX mouse with shRNA-vGAT only (**b**) or shRNA-vGAT+training (**c**) at week 6. **d**. Color-coded representation of gait parameters showing shRNA-vGAT effects (|factor loading|>0.5) represented in PC1. Parameters are grouped into functional clusters. **e**. Examples of kinematic parameters with additive effects of daily training combined with shRNA-vGAT (n=6 each for no intervention, training only, shRNA-vGAT only, and n=7 for shRNA-vGAT+training).

After an intraspinal injection of AAV-floxed-shRNA-vGAT in *vGlut2*^*cr*e^::*Tau*^*LSL -SynGFP*^ mice at P30, we performed a complete transection at P60 and assessed stepping capability 6 weeks after injury (Fig. 8b). All mice with shRNA-vGAT injection exhibited a few spontaneous consecutive steps, albeit limited and uncoordinated, compared to almost no steps by untreated mice (Fig. 8c,d and Extended Data Fig. 7a,b). These data suggest that the treatment influences recovery, although only partially and variably. Lack of complete recovery is not surprising as a severe injury leads to other circuit changes independent of NT phenotype switch or maintenance^15,16,38–40^. Therefore, we then combined this intervention with weight-supported locomotor training known to ameliorate such maladaptive circuit-wide plasticity.

We subjected subsets of adult cSTX mice with or without shRNA-vGAT intervention to daily treadmill training of 20 minutes for 4 weeks (Fig. 8e). To train mice after adult injury, we used wide-spectrum serotonergic receptor agonists that acutely and temporally enable alternating weight-bearing stepping in rodents with complete spinal cord transection^2,3^. These agonists elevate the excitability of the spinal cord and allow training without any long-term effects^41^.

**Extended Data Fig. 8:**
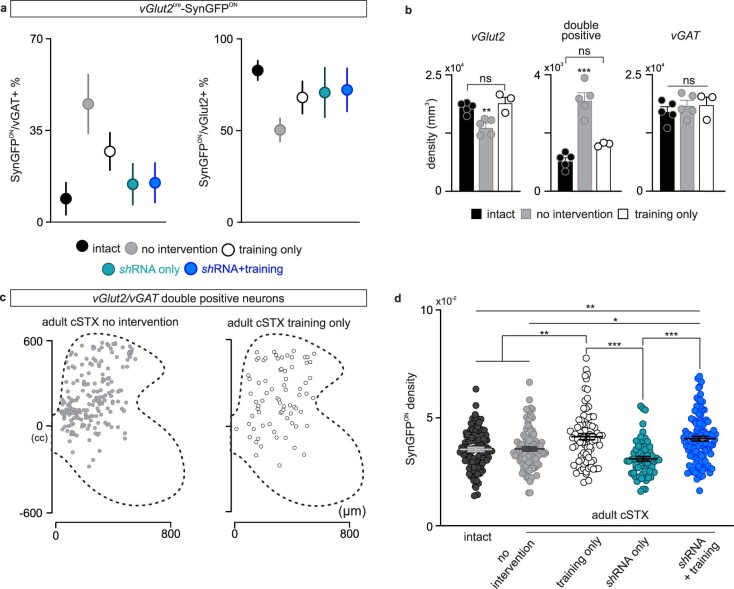
Locomotor training alone after adult injury facilitates circuit reorganization mediated by subpopulations of vGlut2^ON^ interneurons. **a**. NT phenotype of SynGFP^ON^ synaptic terminals with co-labeling of vGAT or vGlut2 antibodies (vGAT^+^/vGlut2^+^, intact n=5, no intervention n=5, training only n=4, shRNA-vGAT only n=6, shRNA-vGAT+training n=7). Intact and adult cSTX spontaneous data is identical to Fig. 2d. Each data point represents an average of all mice, with the error bar indicating ± 1 SD of SynGFP^ON^/vGAT^+^ or SynGFP^ON^/vGlut2^+^ input density per MN (n=10 reconstructed MN per mouse). **b**. Cell density of singular *vGlut2*, double-positive and singular *vGAT* (intact n=5, adult cSTX with no intervention n=5, and adult cSTX trained n=3, 2–3 sections per spinal cord). Intact and adult cSTX untrained data is identical to Fig. 3c). The training effect is also obvious at the transcript level, reducing double-positive cells back to intact levels. (**p<0.01, ***p<0.001, one-way ANOVA with Tukey’s post-hoc test). Mean percentages; intact: *vGlut2* 48%, double-positive 3%, *vGAT* 49%; adult cSTX untrained: *vGlut2* 41%, double-positive 9%, *vGAT* 50%; adult cSTX trained: *vGlut2* 48%, double-positive 4%, *vGAT* 48%. **c**. Location of *vGlut2*/*vGAT* double-positive cells in adult cSTX trained spinal cord (6x16 µm thick section). This reduction was particularly prominent at the dorsal and intermediate lamina. Adult cSTX with no intervention is identical to Fig. 3b**. d**. Daily training leads to increase in density of SynGFP^ON^ terminals. This result indicates that daily training facilitates synaptic sprouting of SynGFP^ON^ terminals (>10 reconstructed motor neurons per neurotransmitter-type, per mouse, intact n=5, adult cSTX with no intervention n=5, adult cSTX training only n=4, adult cSTX shRNA only n=3 and adult cSTX shRNA+training n=4). Intact and adult cSTX with no intervention data is identical to Fig. 2d (*p<0.05, **p<0.01, ***p<0.001, one-way ANOVA with Tukey’s post-hoc test). Error bars: SEM.

After 4 weeks of training, we quantified spontaneous locomotor capacity without serotonergic agonists. Like the shRNA-only group, the locomotor ability of the individual mouse was highly variable (Extended Data Fig. 7c). Nonetheless, PC1 captured shRNA-vGAT effects of both groups with and without training (34% of explained variance), where training further enhanced shRNA-vGAT effects (Fig. 8f and Supplementary Video 4). Extraction and grouping of parameters highly correlated with PC1 demonstrate that shRNA-vGAT combined with daily training significantly reduced the percentage of dragging and improved joint oscillation, measures associated with locomotor recovery^41,42^ (Fig. 8g, Extended Data Fig. 7d,e and Supplementary Video 4). These shRNA-treated groups exhibited a reduction and increase in immunolabeled vGAT^+^ or vGlut2^+^ expression, respectively, compared to the group without any intervention after adult injury (Extended Data Fig. 8a,b). Although training alone does not improve locomotor ability after adult injury, a finding that is consistent with previous studies^2^ (Fig. 8f,g), we found that training alone partially prevented NT phenotype switch—that is, fewer dorsal and intermediate excitatory interneurons expressing vGAT compared to no intervention (Extended Data Fig. 8a–c). Furthermore, training facilitated vGlut2^ON^ synaptic sprouting, a circuit reorganization signature that we detected after neonatal injury (Extended Data Fig. 8d), which could be a potential contributing factor to the added recovery observed in the shRNA+training group.

Together, our results demonstrate that attenuating the injury-induced vGAT expression by excitatory neurons alone promotes locomotor pattern expression, and a combination of long-term locomotor training further facilitates distinct aspects of locomotor recovery after adult injury (Fig. 8h).

## Discussion

Age-dependent plasticity of the CNS enables the spinal cord to ‘learn’ to walk without the brain when an injury is imposed shortly after birth. In contrast, the same injury to an adult leads to permanent paralysis. Our study reveals that age of injury-dependent locomotor circuit plasticity operates with remarkable cell type specificity, in which defined subsets of excitatory, but not inhibitory, interneurons shift the EI synaptic input ratio to MNs. Two distinct activity-dependent mechanisms, NT phenotype and synaptic sprouting, establish the age of injury-dependent, opposing pre-motor synaptic profiles with high inhibition after adult injury in contrast to increased vGlut2 synaptic input after neonatal injury. Virus-mediated opposing circuit manipulations after neonatal or adult injury suggest that the EI input ratio from pre-motor interneurons to MNs shapes locomotor ability after spinal cord injury (Fig. 8h). We discuss how these findings advance understanding of spontaneous circuit reorganization after a severe spinal cord injury, by which age of injury, cell type and activity influence the recovery process.

We found that the NT phenotype switch is one of the signatures of spinal cord plasticity, a previously unreported mechanism in the mammalian spinal cord. Studies in spinal-cord-injured cats and rodents demonstrated that modulation of inhibitory transmission is critical for improving motor output and training outcome^13,14,18,19^. These studies, however, left open the question of the source of inhibitory drive to MNs in this activity-regulated process. Unexpectedly, we found that the synaptic source of vGAT input to MN after injury was not derived from inhibitory interneurons but, rather, from vGlut2^ON^ interneurons switching NT phenotype.

We uncovered that a subset of spinal interneurons exhibits flexible NT phenotype, depending on the age of injury and activity level. NT phenotype switch occurs in many species, including *Caenorhabditis* *elegans*, zebrafish, *Xenopus* and rodents, during development and mature CNS^31,43–45^. Although some neurons co-express and co-release two distinct NTs from the same synaptic terminal^46–48^, some release two NTs selectively from different terminals^34,35^. Our findings on spinal interneurons are consistent with the latter, in which a subset of excitatory spinal interneurons co-expresses *vGlut2* and *vGAT* transcripts at the cell body, yet there was no accumulation of both NTs at synaptic terminals.

What is the implication of having neurons with flexible, activity-dependent NT phenotype expression in the spinal cord? We found a low level of *vGlut2* and *vGAT* transcript co-expression at the cell body in the intact condition and a small number of vGAT^+^ terminals in genetically labeled excitatory interneurons. This finding indicates that there are populations of neurons capable of adjusting NT expression under normal circumstances. An increase in locomotor activity leading to NT phenotype switch of defined midbrain neurons in intact mice^23^ suggests that motor activity may also modulate the baseline level of NT phenotypes in the intact spinal cord, like for motor learning. Likewise, such flexibility offers an opportunity to harness NT phenotype switches as a therapeutic approach to augment rehabilitative training and prevent maladaptive circuit functions after a severe injury^15,38^.

To date, mechanisms that underlie the closure of the enhanced plasticity time window after neonatal injury remain unknown. The sensitive period for achieving proficient locomotion without the brain closes around P15 in rodents^49^, a timepoint when a severe injury leads to a diverging outcome of proficient locomotion or paralysis. This time window corresponds to when corticospinal inputs reach the lumbar spinal cord, and brainstem-descending pathways establish mature connectivity patterns^50–52^. It is, therefore, possible that cell-intrinsic plasticity of spinal interneurons changes fundamentally around this time to facilitate the integration of descending inputs and local circuits to enable the execution of proficient motor programs. Our work revealed that baseline PA activity is necessary to maintain glutamatergic NT phenotype by excitatory interneurons after neonatal injury, which we demonstrate to be an essential mechanism to learn to walk without the brain. However, PAs do not regulate gene expression of NTs after adult injury in a similar manner. Instead, spinal circuits injured as an adult require intense, movement-directed somatosensory activation to partially prevent the gain of vGAT phenotype by glutamatergic interneurons and for synaptic sprouting. In support of this idea that cell-intrinsic plasticity changes around P15, the end of the sensitive period for the age of injury-dependent locomotor circuit plasticity coincides with a marked shift in gene expression pattern throughout the CNS^53^.

We also found that subpopulations of excitatory interneurons and their synaptic connectivity profiles define locomotor capacity after complete spinal cord injury. In contrast, NT phenotype and connectivity from the inhibitory interneurons to MNs are hardwired and unaffected by injury or age of injury. Of the three excitatory interneuron populations examined, dorsal and intermediately located interneurons were much more plastic than ventral interneurons. Based on findings from PA ablation, activity-dependent NT phenotype re-specification and sprouting, we speculate that spatial positioning of these neurons—that is, their access to PA input—is a critical factor in the observed cell-type-specific plasticity. These dI3/5 and V2 interneurons reside along the dorsoventral and mediolateral axes with abundant PA innervation, whereas most V3 neurons sit medioventrally where PAs innervate sparsely. Recent transcriptomic analysis revealed cell-type-specific response to injury along the dorsoventral axis^54^. These findings point to the possible importance of spatial positioning within the spinal cord.

Consequently, the identity and functional roles of more plastic excitatory interneurons in locomotion, per se, may be a fortuitous secondary factor to facilitate locomotion without the brain. For instance, defined subpopulations of V2 neurons are responsible for either locomotor rhythm or patterns with abundant interconnections within each functional group^55,56^. In contrast, the roles of dI3 and dI5 interneurons in locomotion are unknown. *Tlx3* is a selector gene that determines a glutamatergic over GABAergic neuron cell fate during development^57^. NT phenotype switch in the *Tlx3* population is likely unrelated to the function of *Tlx3* as we study them in adulthood when *Tlx3* is expressed at a low level. Nonetheless, our work suggests that the vGlut2 synaptic drive from these neurons to MNs is critical for regulating locomotion without the brain. Roles of V3 interneurons are yet to be defined.

The age of injury-dependent EI input balance from spinal interneurons is consistent with the need for increased excitability for movement generation after injury^12–14^. However, the finding that benefits locomotor recovery may seem paradoxical to maladaptive hyperexcitability observed after spinal cord injury^15,16,40^. Therefore, identifying other sources of MN excitation and the extent of their contribution to recovery and maladaptation remain essential. In addition to pre-motor neurons, MN physiology after a spinal cord injury represents a collective shift in excitability of MN^15,16^ and vGlut1^ON^ input of muscle spindle Ia afferents and their regulation by GABApre neurons. They are known culprits to mediate maladaptive state of hyperexcitability leading to muscle spasticity^58–62^. It remains unclear whether and how distinct local circuit modules interact to achieve the coordinated activation and inhibition of the local circuits in movement-matched sequence and vigor after neonatal injury or shRNA-treated mice after adult injury.

In summary, we identified that NT phenotype and synaptic sprouting of specific populations of excitatory interneurons serve as a neuronal basis of age of injury-dependent locomotor capacity after severe spinal cord injury. This finding stresses the importance of targeting defined neurons in rehabilitative strategies after spinal cord injury. Together with recent studies in humans demonstrating benefits of increased excitability of the spinal cord temporarily and acutely with electrical stimulation^4–6^ and rodent models to modulate EI balance of circuits^42,63^, enacting optimal EI balance below lesion may be achieved in part by regulation of interneurons. Similar concepts may apply to other traumatic CNS disorders that highly rely on plasticity attributed to the age of patients and circuit-specific targeting to regulate EI balance for locomotor recovery.

## Methods

### Mouse genetics

Wild-type (C57Bl6), *PV*^cre^ (JAX stock no. 008069), *Advillin*^iDTR^ (ref. ^64^), *vGlut2*^cre^ (JAX stock no. 028863), *vGAT*^cre^ (JAX stock no. 016962), *Tlx3*^cre^ (041158-UCD), *Shox2*^cre^ (ref. ^55^), *Sim1*^cre^ (ref. ^65^), *Rosa26*^*LSL-tdTomato*^ (JAX stock no. 007908) and *Tau*^*LSL-nlsLacZ-SynGFP-INLA*^ (ref. ^29^) mouse strains were maintained on a mixed genetic background (129/C57Bl6). Adult mice of both sexes, aged 2–6 months, were used for all experiments. Mice were group housed, and all mice were kept on a 12-hour light/dark cycle in a facility where temperature was kept at 22 ± 2 °C and ~50% humidity. All animal procedures were conducted in accordance with Belgian regulations and were approved by the institutional ethical committee of KU Leuven.

### Spinal cord injury and daily care

For neonatal complete spinal cord transection, 5-day-old pups were anesthetized with ice, and muscles were separated along the midline to expose the vertebral column. Laminectomy was performed using forceps at spinal segment ∼T10, and the spinal cord was completely transected using micro-scissors. After surgery, mice were placed in a heat chamber until fully awake. All other surgical procedures were performed under aseptic conditions and full general anesthesia with isoflurane in oxygen-enriched air (1–2%). Adult mice underwent a mid-dorsal skin incision to access the spinal cord, and a laminectomy was made over the spinal segment T10. A complete spinal cord transection was performed using micro-scissors. Analgesia (Metacam, Boehringer Ingelheim, 0.5 mg ml^−1^) was provided after surgery, and antibiotics (Cefazoline, Sandoz, 5 mg kg^−1^) were provided for 3 days after surgery. Mice were housed on a 12-hour light/dark cycle with ad libitum access to food and water and cared for twice daily with manual bladder voiding. Complete transections were confirmed postmortem by visual inspection of the spinal cord.

### Kinematic training and recordings

Bipedal locomotor kinematic recordings were performed using the high-speed motion capture system Vicon (Vicon Motion Systems). Ten infrared cameras (200 Hz) were used to detect 3-mm reflective markers attached bilaterally on the mice overlying the iliac crest, the greater trochanter (hip), the lateral condyle (knee), the malleolus (ankle) and the base of the metatarsal phalangeal joint. Positions of the markers were reconstructed offline in three dimensions (3D) using Vicon Nexus software (version 2.12). The hindlimb was modeled as interconnected segments, and joint angles were calculated accordingly. Parameters (Supplementary Table 1) describing gait timing, joint kinematics, limb endpoint trajectory and stability were computed for each gait cycle using custom-written MATLAB scripts and according to methods detailed previously^28^. Locomotor training started 2 weeks after adult spinal transection and occurred for 20 minutes per day for 4 weeks on a motorized treadmill with body weight support (Robomedica) at 5–15 cm s^−1^, after intraperitoneal injection of monoaminergic agonists, consisting of 5-HT1A/7 agonist 8-Hydroxy-DPAT hydrobromide (Sigma-Aldrich, H140, 0.375 mg kg^−1^) and 5-HT2A/C agonist Quipazine (Sigma-Aldrich, Q1004, 0.125 mg kg^−1^). Neonatally injured or adult injured mice with muscle atrophy, incomplete spinal cord injury or occasional spinal cord degeneration were excluded from the study.

### Kinematic analysis

Step 1: Locomotor patterns are recorded using high-resolution cameras.

Step 2: Custom-written MATLAB scripts are used to reconstruct kinematic parameters. All variables computed for each task are specified in Supplementary Table 1. Approximately 15–20 steps were extracted per mouse.

Step 3: All computed variables were averaged for each mouse independently. All body-size-dependent parameters were normalized according to body weight. The matrix combining all mean values of variables from all mice of analysis was then subjected to a PC analysis. For this purpose, we used the correlation method, which adjusts the mean of the data to 0 and the standard deviation to 1. A new set of synthetic uncorrelated variables—that is, the PCs—each explains the maximum possible amount of variance.

Step 4: The new coordinates of gait patterns along each PC, termed PC scores, are extracted for each mouse. PC scores represent gait patterns in the ‘denoised’ PC space to visualize differences between experimental conditions.

Step 5: PC scores are averaged for each experimental condition and represented in histogram plots to identify the type of information differentiated along each PC axis.

Step 6: Each PC is a linear combination of the original parameters with appropriate weights, termed ‘factor loadings’. The values of factor loadings range from −1 to 1 and correspond to correlations between original parameters and a given PC.

Step 7: Factor loadings with a high value (|factor loading| > 0.5) are extracted, color-coded based on their correlation value and regrouped into functional clusters.

Locomotor analysis after shRNA injection. For the shRNA+training group, mice underwent daily locomotor training starting from 2 weeks after lesion for 4 weeks. Kinematic recordings were performed at week 5 and week 6 for all four groups (no intervention, training only, shRNA only and shRNA+training). The number of consecutive steps, dorsal and plantar steps combined and bouts were quantified over one recording session (30 seconds) from either week 5 or week 6. Dragging is defined as a contact of the dorsal surface of the foot to the ground during the swing phase. After kinematic reconstruction, each step cycle is manually inspected by an experimenter with 100-Hz digital camera recordings to confirm foot ON/OFF segments.

### DTX delivery for PA ablation

DTX (Sigma-Aldrich, D0564) was injected intraperitoneally for adult mice (100 mg kg^−1^) and subcutaneously for P5 mice (100 µg kg^−1^) following an established protocol^30^. To verify ablation efficiency, we determined vGlut1^ON^ afferent density postmortem. Inclusion criteria were set at >50% ablation after normalization compared to intact conditions.

### Virus production

G-deleted rabies with fluorescent Tag was amplified and purified from local viral stocks following established protocols^66^. All AAVs used in this study were of genomic titers >1 × 10^13^.

### Intraspinal injections

Under deep general anesthesia using either isoflurane enriched with oxygen (1–2%) for adult mice or hypothermia for P5 mice, a mid-dorsal skin incision and laminectomy were performed to expose the spinal cord. The intraspinal injection was made using a pulled calibrated glass pipette (World Precision Instruments) by multiple short pulses (3 ms, 0.5 Hz) using a picospritzer (Parker Hannifin).

### Virus-mediated manipulation

#### Neonatal injured mice

A bilateral intraspinal virus injection of AAV-DIO-vGAT-Tag or AAV-DIO-Tag in lumbar segments of *vGlut2*^cre^::*Tau*^*LSL-lacZ-SynGFP-INLA*^ mice was performed concomitantly with a T10 complete transection of the spinal cord on neonatal mice at P5. We aimed to deposit the virus in the dorsal/intermediate lamina, where the NT phenotype switch occurs. Mice underwent kinematic recordings around P60. Virus infection efficiency was determined postmortem, with inclusion criteria set at >95% survival of genetically marked vGlut2^ON^ interneurons, compared to a cohort of mice without intraspinal injection, and >90% of virus-tagged neurons are in the dorsal or intermediate lamina where the double-positive neurons reside after adult injury or P5 cSTX+PA^DTX^. In addition, we only included mice that showed the vGAT gradient ranges from the highest vGAT^ON^/SynGFP^ON^ terminal value of P5 cSTX+vGAT to the lowest of P5 cSTX+control (25–65%, cyan to magenta), within ±2 standard deviations of the % SynGFP^ON^/vGAT^+^ computed from individual MNs in the adult cSTX group (mean 45%, range 25–65%; Fig. 2d).

#### Adult injured mice

A bilateral intraspinal virus injection of AAV-floxed-*sh*RNA-vGAT-Tag was performed in lumbar segments of ~3-week-old *vGlut2*^cr*e*^::*Tau*^*LSL-lacZ-SynGFP-INLA*^ mice, 2 weeks before a T10 complete spinal transection. After 2 weeks of recovery, maximum consecutive steps were counted over one recording session (30 seconds) at week 5 or week 6. For the shRNA+training group, mice underwent daily locomotor training for 4 weeks, and kinematic recordings were obtained. Virus infection efficiency was determined postmortem, with inclusion criteria set at <30% mean expression of vGAT^+^ at SynGFP^ON^ terminals. A subset of mice underwent a mock injection before the injury as a control.

#### Descending circuit tracing with G-deleted rabies

Using isoflurane anesthesia, mice underwent laminectomy to expose the surface of the spinal cord. A pulled calibrated glass pipette (Drummond Scientific) was used for local application of 100 nl of virus by multiple short pulses (3 ms, 0.5 Hz) using a picospritzer (Parker Hannifin). After surgery, mice were administered Metacam (5 mg kg^−1^) subcutaneously for analgesia. Mice were perfused for analysis 4 days after injections.

### Histological analysis

We used spinal cord tissue >1 month after injury for both P5 and adult cSTX groups. To make sure that age of tissue per se is not the driving factor for *vGlut2/vGAT* gene expression and synaptic profile connectivity, we confirmed that quantification obtained from P5 cSTX tissue at an older age (that is, age-matched to adult cSTX at ~P75–90) does not differ from P5 cSTX at ~P35.

### In situ hybridization

In situ detection was performed on 16-µm-thin cryosections of fresh frozen spinal cord sections using RNAscope Fluorescent Multiplex Assay (Advanced Cell Diagnostics) with custom-designed probes (RNAscope Probe-Mm-Slc17a6 (vGlut2) 319171, RNAscope Probe-Mm-Slc32a1-C2 (vGAT) 319191-C2 and RNAscope Probe-Mm-TdTomato-C3 317041-C3). Tissue sections were labeled with DAPI (Advanced Cell Diagnostics, 320858).

### Immunohistochemistry

All mice were deeply anesthetized with ketamine (Nimatek, Dechra, CNK:3120–060, 100 mg kg^−1^, intraperitoneally) and xylazine (Xyl-M, Livestock Pharma, BE-V170581, 5 mg kg^−1^, intraperitoneally) and perfused with 4% paraformaldehyde. All collected tissue was cryoprotected in 30% sucrose/PBS and cut on a cryostat (spinal cord: 40–50-µm transverse sections; brain: 80-µm sagittal sections). Floating tissue sections were incubated with antibodies in individual wells and slide-mounted for imaging. Antibodies used in this study were as follows: chicken anti-β-galactosidase (1:5,000, Abcam, AB9361); chicken anti-GFP (1:500, Molecular Probe, A10262); goat anti-ChAT (1:1,000, Chemicon, AB144P); guinea pig anti-gephyrin (1:1,000, SySy, 147–318); guinea pig anti-vGlut1 (1:20,000, Chemicon, AB5905); mouse anti-glycine receptor alpha 1 (GlyRα1) (1:2,000, SySy, 146–111); mouse anti-NeuN (1:1,000, Millipore, MAB377); mouse anti-vesicular GABA transporter (vGAT) (1:500, SySy, 131–011); mouse anti-vesicular glutamate transporter 2 (vGlut2) (1:500, Merck Millipore, MAB5504); rabbit anti-GABA (1:4,000, Sigma-Aldrich, A2052); rabbit anti-GFP (1:5,000, Thermo Fisher Scientific, A11122); rabbit anti-vesicular GABA transporter (vGAT) (1:2,000, Merck Millipore, AB5062P); and rabbit anti-vesicular glutamate transporter 2 (vGlut2) (1:500, SySy, 135–403). Fluorophore-coupled secondary antibodies were obtained from Jackson Laboratory and used at 1:1,000: Alexa Fluor 488 donkey anti-chicken (703-545-155); Alexa Fluor 488 donkey anti-guinea pig (706-545-148); Alexa Fluor 488 donkey anti-mouse (715-545-150); Cy3 donkey anti-goat (705-165-147); Cy3 donkey anti-guinea pig (706-165-148); Cy3 donkey anti-mouse (715-165-150); Cy3 donkey anti-rabbit (711-165-152); Alexa Fluor 647 donkey anti-goat (705-605-147); Alexa Fluor 647 donkey anti-mouse (715-605-150); and Alexa Fluor 647 donkey anti-rabbit (711-605-152).

### 3D digital brain and spinal cord reconstructions

All brain and spinal cord images were acquired using a slide scanner (Nikon, ×40 objective) or a confocal (Zeiss, ×10 objective) microscope and aligned using ImageJ TrakEM. Surfaces were automatically detected using the Imaris Surface Detection module. Rabies^ON^ neurons were identified manually using Imaris (Bitplane) spot detection and color-coded according to their location based on Paxinos and Franklinʼs mouse brain atlas. Coordinates of rabies-marked neurons were assigned manually.

### RNAscope co-localization analysis

Spinal cord pictures were acquired using a slide scanner microscope (Nikon, ×40 objective, 0.7-µm step size). Neurons were categorized into three different categories using Imaris. Neurons were considered *vGlut2* or *vGAT* only when the cell contained >8 transcripts of *vGlut2* or *vGAT*. Neurons were considered double-positive when the cell contained >8 transcripts of one NT and >4 transcripts of the other NT. The number of neurons per category was normalized to the volume of the gray matter. Alignments were normalized using central canal coordinates. Poorly prepared samples were excluded from the study (DAPI^+^ cell density <40,000 mm^3^).

### Synaptic quantification analysis

High-resolution analysis of synaptic input to interneurons and MNs was acquired using a confocal microscope (Zeiss, ×63 objective, 0.2-µm step size). Imaris was used for the quantification of interneuronal input to ChAT^ON^ MNs. Synaptic density was computed by the number of synaptic terminals normalized to the surface area of ChAT^ON^ MNs. The ROUT method was used for the identification and removal of outliers.

### β-galactosidase analysis

Images were acquired using a confocal microscope (Zeiss, ×10 objective, 1-µm step size). Alignment, quantification and extraction of coordinates were performed with ImageJ.

### Statistics and reproducibility

All statistical analyses were performed and plotted using R (version 3.5), MATLAB (version 2014a or version 2018a) or GraphPad Prism (version 8.0). The means of different data distributions were compared using two-sided unpaired *t*-tests (Figs. 1f, 2c,d, 3c, 4a and 8d and Extended Data Figs. 4b and 5e), one-way ANOVA with Tukey’s post hoc analysis (Figs. 4b, 5c, 6e, 7c and 8f,g and Extended Data Figs. 3b, 4e, 7e and 8b,d) and correlation analysis (Fig. 7d). Significance level is defined as follows for all analyses performed: *P < 0.05, **P < 0.01, ***P < 0.001. Power calculation was assessed to be all over the desired power of >0.80. All used high-resolution images are representative of the described experiments. Data distribution was assumed to be normal, but this was not formally tested. Except for random subsampling of behavioral and anatomical data, randomization was not used during analysis. Animals (within genotype pools) were randomly assigned to experimental groups. Data collection and analysis were not performed blinded to the conditions of the experiments. Animals from different experimental groups were age-matched. A subset of animals was excluded from experiments post hoc (see above subsections for exclusion criteria).

### Reporting Summary

Further information on research design is available in the Nature Research Reporting Summary linked to this article.

## Online content

Any methods, additional references, Nature Research reporting summaries, source data, extended data, supplementary information, acknowledgements, peer review information; details of author contributions and competing interests; and statements of data and code availability are available at 10.1038/s41593-022-01067-9.

## Supplementary information

Supplementary Information Supplementary Table 1

Reporting Summary

Supplementary Video 1 Intact and P5 cSTX mice show proficient weight-supported bipedal locomotion (15 cm s^−1^) compared to dragging adult cSTX mice

Supplementary Video 2 A complete spinal cord retransection of P5 cSTX mice does not alter locomotor proficiency

Supplementary Video 3 Virus-mediated vGAT expression by vGlut2^ON^ neurons after neonatal injury deteriorates the ability to walk without the brain

Supplementary Video 4 Virus-mediated vGAT suppression by vGlut2^ON^ neurons after adult injury facilitates stepping ability

## Data Availability

Data from this study are available from the corresponding author upon reasonable request.

## Code availability

Custom code used in this study is available from the corresponding author upon reasonable request.

## References

[1] Alluin O, Delivet-Mongrain H, Rossignol S (2015). Inducing hindlimb locomotor recovery in adult rat after complete thoracic spinal cord section using repeated treadmill training with perineal stimulation only. J. Neurophysiol..

[2] Fong AJ (2005). Spinal cord-transected mice learn to step in response to quipazine treatment and robotic training. J. Neurosci..

[3] Courtine G (2009). Transformation of nonfunctional spinal circuits into functional states after the loss of brain input. Nat. Neurosci..

[4] Angeli CA (2014). Altering spinal cord excitability enables voluntary movements after chronic complete paralysis in humans. Brain.

[5] Gill ML (2018). Neuromodulation of lumbosacral spinal networks enables independent stepping after complete paraplegia. Nat. Med..

[6] Harkema S (2011). Effect of epidural stimulation of the lumbosacral spinal cord on voluntary movement, standing, and assisted stepping after motor complete paraplegia: a case study. Lancet.

[7] Cummings JP, Bernstein DR, Stelzner DJ (1981). Further evidence that sparing of function after spinal cord transection in the neonatal rat is not due to axonal generation or regeneration. Exp. Neurol..

[8] Stelzner DJ, Ershler WB, Weber ED (1975). Effects of spinal transection in neonatal and weanling rats: survival of function. Exp. Neurol..

[9] Tillakaratne NJK (2010). Functional recovery of stepping in rats after a complete neonatal spinal cord transection is not due to regrowth across the lesion site. Neuroscience.

[10] Norreel J-C (2003). Reversible disorganization of the locomotor pattern after neonatal spinal cord transection in the rat. J. Neurosci..

[11] Petruska JC (2007). Changes in motoneuron properties and synaptic inputs related to step training after spinal cord transection in rats. J. Neurosci..

[12] Barbeau, H. J., Julien, C. & Rossignol, S. The effects of clonidine and yohimbine on locomotion and cutaneous reflexes in the adult chronic spinal cat. *Brain Res.* **437**, 83–96 (1987). 10.1016/0006-8993(87)91529-0 3427484

[13] Robinson GAG, Goldberger MEM (1985). Interfering with inhibition may improve motor function. Brain Res..

[14] de Leon RD, Tamaki H, Hodgson JA, Roy RR, Edgerton VR (1999). Hindlimb locomotor and postural training modulates glycinergic inhibition in the spinal cord of the adult spinal cat. J. Neurophysiol..

[15] Boulenguez P (2010). Down-regulation of the potassium-chloride cotransporter KCC2 contributes to spasticity after spinal cord injury. Nat. Med..

[16] Murray KC (2010). Recovery of motoneuron and locomotor function after spinal cord injury depends on constitutive activity in 5-HT2C receptors. Nat. Med..

[17] Ichiyama RM (2011). Locomotor training maintains normal inhibitory influence on both alpha- and gamma-motoneurons after neonatal spinal cord transection. J. Neurosci..

[18] Tillakaratne N (2000). Increased expression of glutamate decarboxylase (GAD67) in feline lumbar spinal cord after complete thoracic spinal cord transection. J. Neurosci. Res..

[19] Tillakaratne NJK (2002). Use-dependent modulation of inhibitory capacity in the feline lumbar spinal cord. J. Neurosci..

[20] Hrvatin S (2017). Single-cell analysis of experience-dependent transcriptomic states in the mouse visual cortex. Nat. Neurosci..

[21] Spiegel I (2014). Npas4 regulates excitatory-inhibitory balance within neural circuits through cell-type-specific gene programs. Cell.

[22] Meng D, Li HQ, Deisseroth K, Leutgeb S, Spitzer NC (2018). Neuronal activity regulates neurotransmitter switching in the adult brain following light-induced stress. Proc. Natl Acad. Sci. USA.

[23] Li, H.-Q. & Spitzer, N. C. Exercise enhances motor skill learning by neurotransmitter switching in the adult midbrain. *Nat. Commun.* **11**, 2195 (2020). 10.1038/s41467-020-16053-7 PMC7198516 32366867

[24] Brown AG (1981). Organization of the Spinal Cord.

[25] Eccles JC, Eccles RM, Lundberg A (1957). The convergence of monosynaptic excitatory afferents on to many different species of alpha motoneurones. J. Physiol..

[26] Windhorst U (2007). Muscle proprioceptive feedback and spinal networks. Brain Res. Bull..

[27] Bui TV (2013). Circuits for grasping: spinal dI3 interneurons mediate cutaneous control of motor behavior. Neuron.

[28] Walton KD, Lieberman D, Llinas A, Begin M, Llinás RR (1992). Identification of a critical period for motor development in neonatal rats. Neuroscience.

[29] Tripodi M, Stepien AE, Arber S (2011). Motor antagonism exposed by spatial segregation and timing of neurogenesis. Nature.

[30] Takeoka A, Arber S (2019). Functional local proprioceptive feedback circuits initiate and maintain locomotor recovery after spinal cord injury. Cell Rep..

[31] Dulcis D, Spitzer NC (2008). Illumination controls differentiation of dopamine neurons regulating behaviour. Nature.

[32] Nishimaru H, Restrepo CE, Ryge J, Yanagawa Y, Kiehn O (2005). Mammalian motor neurons corelease glutamate and acetylcholine at central synapses. Proc. Natl Acad. Sci. USA.

[33] Mentis GZ (2005). Noncholinergic excitatory actions of motoneurons in the neonatal mammalian spinal cord. Proc. Natl Acad. Sci. USA.

[34] Zhang S (2015). Dopaminergic and glutamatergic microdomains in a subset of rodent mesoaccumbens axons. Nat. Neurosci..

[35] Root DH (2020). Distinct signaling by ventral tegmental area glutamate, GABA, and combinatorial glutamate- GABA neurons in motivated behavior. Cell Rep..

[36] Purves D, Lichtman JW (1980). Elimination of synapses in the developing nervous system. Science.

[37] Alaynick WA, Jessell TM, Pfaff SL (2011). SnapShot: spinal cord development. Cell.

[38] Beauparlant J (2013). Undirected compensatory plasticity contributes to neuronal dysfunction after severe spinal cord injury. Brain.

[39] Ryge J (2010). Transcriptional regulation of gene expression clusters in motor neurons following spinal cord injury. BMC Genomics.

[40] Bellardita C (2017). Spatiotemporal correlation of spinal network dynamics underlying spasms in chronic spinalized mice. eLife.

[41] Bui, T. V., Stifani, N., Akay, T. & Brownstone, R. M. Spinal microcircuits comprising dI3 interneurons are necessary for motor functional recovery following spinal cord transection. *eLife* **5**, e21715 (2016). 10.7554/eLife.21715 PMC5218533 27977000

[42] Chen B (2018). Reactivation of dormant relay pathways in injured spinal cord by KCC2 manipulations. Cell.

[43] Serrano-Saiz E (2013). Modular control of glutamatergic neuronal identity in C-elegans by distinct homeodomain proteins. Cell.

[44] Bertuzzi M, Chang W, Ampatzis K (2018). Adult spinal motoneurons change their neurotransmitter phenotype to control locomotion. Proc. Natl Acad. Sci. USA.

[45] Dulcis D, Jamshidi P, Leutgeb S, Spitzer NC (2013). Neurotransmitter switching in the adult brain regulates behavior. Science.

[46] Shabel SJ, Proulx CD, Piriz J, Malinow R (2014). Mood regulation. GABA/glutamate co-release controls habenula output and is modified by antidepressant treatment. Science.

[47] Saunders A, Granger AJ, Elife BS (2015). Corelease of acetylcholine and GABA from cholinergic forebrain neurons. eLife.

[48] Hnasko TS, Edwards RH (2012). Neurotransmitter corelease: mechanism and physiological role. Annu. Rev. Physiol..

[49] Weber ED, Stelzner DJ (1977). Behavioral effects of spinal cord transection in the developing rat. Brain Res..

[50] Donatelle JM (1977). Growth of the corticospinal tract and the development of placing reactions in the postnatal rat. J. Comp. Neurol..

[51] Bregman BS (1987). Development of serotonin immunoreactivity in the rat spinal cord and its plasticity after neonatal spinal cord lesions. Brain Res..

[52] Gianino SS (1999). Postnatal growth of corticospinal axons in the spinal cord of developing mice. Brain Res. Dev. Brain Res..

[53] Tan L (2021). Changes in genome architecture and transcriptional dynamics progress independently of sensory experience during post-natal brain development. Cell.

[54] Matson, K. J. E. et al. A single cell atlas of spared tissue below a spinal cord injury reveals cellular mechanisms of repair. Preprint at https://www.biorxiv.org/content/10.1101/2021.04.28.441862v1 (2021).

[55] Dougherty KJ (2013). Locomotor rhythm generation linked to the output of spinal shox2 excitatory interneurons. Neuron.

[56] Ha, N. T. & Dougherty, K. J. Spinal Shox2 interneuron interconnectivity related to function and development. *eLife* **7**, e42519 (2018). 10.7554/eLife.42519 PMC6333440 30596374

[57] Cheng L (2004). *Tlx3* and *Tlx1* are post-mitotic selector genes determining glutamatergic over GABAergic cell fates. Nat. Neurosci..

[58] Hultborn, H. Changes in neuronal properties and spinal reflexes during development of spasticity following spinal cord lesions and stroke: studies in animal models and patients. *J. Rehabil. Med.* 46–55 (2003). 10.1080/16501960310010142 12817657

[59] Faist M, Mazevet D, Dietz V, Pierrot-Deseilligny E (1994). A quantitative assessment of presynaptic inhibition of la afferents in spastics: differences in hemiplegics and paraplegics. Brain.

[60] Caron G, Bilchak JN, Côté M-P (2020). Direct evidence for decreased presynaptic inhibition evoked by PBSt group I muscle afferents after chronic SCI and recovery with step-training in rats. J. Physiol..

[61] Nielsen J, Petersen N, Crone C (1995). Changes in transmission across synapses of Ia afferents in spastic patients. Brain.

[62] Thompson FJ, Reier PJ, Lucas CC, Parmer R (1992). Altered patterns of reflex excitability subsequent to contusion injury of the rat spinal cord. J. Neurophysiol..

[63] Brommer B (2021). Improving hindlimb locomotor function by non-invasive AAV-mediated manipulations of propriospinal neurons in mice with complete spinal cord injury. Nat. Commun..

[64] Stantcheva KK (2016). A subpopulation of itch-sensing neurons marked by Ret and somatostatin expression. EMBO Rep..

[65] Zhang Y (2008). V3 spinal neurons establish a robust and balanced locomotor rhythm during walking. Neuron.

[66] Reardon TR (2016). Rabies virus CVS-N2c^ΔG^ strain enhances retrograde synaptic transfer and neuronal viability. Neuron.

