# Supplementary material for: Neurotransmitter phenotype switching by spinal excitatory interneurons regulates locomotor recovery after spinal cord injury
Source: Nat Neurosci. 2022 May 6;25(5):617–29. doi: 10.1038/s41593-022-01067-9 (PMC9076533; doi:10.1038/s41593-022-01067-9)
Supplement: Supplementary file 1 — Supplementary Table 1 [file 41593_2022_1067_MOESM1_ESM.pdf]

nature portfolio

https://doi.org/10.1038/s41593-022-01067-9

Supplementary information

Neurotransmitter phenotype switching by spinal excitatory interneurons regulates locomotor recovery after spinal cord injury

In the format provided by the authors and unedited

		TEMPORAL FEATURES OF GAIT
A	1	Cycle duration (s)
A	2	Cycle velocity (s)
A	3	Stance duration (s)
A	4	Swing duration (s)
A	5	Relative stance duration (% of gait cycle duration)

		INTERLIMB COORDINATION
A	6	Correlation between whole hindlimb oscillations
A	7	Double stance duration (% of gait cycle duration)
A	8	Coordination between stance left-right (% of gait cycle duration)
A	9	Coordination between swing left-right (% of gait cycle duration)

		LIMB ENDPOINT TRAJECTORIES
A	10	Stride length (cm)
A	11	Step length (cm)
A	12	3D limb endpoint path length (cm)
A	13	Maximal backward position of foot (cm)
A	14	Minimum forward position of foot (cm)
A	15	Step height (cm)
B	16	Maximal speed during swing (cm/s)
B	17	Time of maximal velocity during swing (% of gait cycle duration)
B	18	Acceleration at swing onset (cm/s^2)
A	19	Endpoint velocity (cm/s)
A	20	Orientation of the velocity vector at swing onset (deg)

		STABILITY	
A	21	Lateral displacement during swing	Base of support
A	22	Stance width (cm)	Base of support
A	23	Pelvis maximum vertical movement	Base of support
A	24	Pelvis minimum vertical movement	Base of support
A	25	Pelvis vertical movement amplitude	Base of support
A	26	Variability of vertical mid-point hip oscillation	Pelvis position and oscilations
A	27	Variability of medio-lateral mid-point hip oscillation	Pelvis position and oscilations
A	28	Variability of medio-lateral hip rotations	Pelvis position and oscilations
A	29	Forward motion of body center of mass (cm)	Displacement of virtual center of mass
A	30	Lateral motion of body center of mass (cm)	Displacement of virtual center of mass
A	31	Vertical motion of body center of mass (cm)	Displacement of virtual center of mass
A	32	3D motion of body center of mass (cm)	Displacement of virtual center of mass
A	33	Weight support (%)	

		DRAGGING
A	33	Drag duration (s)
A	34	Relative drag duration (% of swing phase duration)

A = Figures 1, 7, 8, S1, S6
A+B = Figures 1, 8, S1, S6

		JOINT ANGLES AND SEGMENTAL OSCILLATIONS	
A	35	Crest oscillations (deg)	Backward
A	36	Thigh oscillations (deg)	Backward
A	37	Leg oscillations (deg)	Backward
A	38	Foot oscillations (deg)	Backward
A	39	Whole limb oscillations (deg)	Backward
A	40	Crest oscillations (deg)	Forward
A	41	Thigh oscillations (deg)	Forward
A	42	Leg oscillations (deg)	Forward
A	43	Foot oscillations (deg)	Forward
A	44	Whole limb oscillations (deg)	Forward
A	45	Hip joint (deg)	Extension
A	46	Knee joint (deg)	Extension
A	47	Ankle joint (deg)	Extension
A	48	Whole limb abduction (deg)	Abduction
B	49	Foot abduction (deg)	Abduction
A	50	Hip joint (deg)	Flexion
A	51	Knee joint (deg)	Flexion
A	52	Ankle joint (deg)	Flexion
A	53	Whole limb adduction (deg)	Adduction
B	54	Foot adduction (deg)	Adduction
A	55	Crest oscillations (deg)	Oscillation amplitudes (Max-Min)
A	56	Thigh oscillations (deg)	Oscillation amplitudes (Max-Min)
A	57	Shank oscillations (deg)	Oscillation amplitudes (Max-Min)
A	58	Foot oscillations (deg)	Oscillation amplitudes (Max-Min)
A	59	Whole limb oscillations (deg)	Oscillation amplitudes (Max-Min)
A	60	Hip joint (deg)	Oscillation amplitudes (Max-Min)
A	61	Knee joint (deg)	Oscillation amplitudes (Max-Min)
A	62	Ankle joint (deg)	Oscillation amplitudes (Max-Min)
A	63	Whole limb medio-lateral oscillation (deg)	Oscillation amplitudes (Max-Min)
B	64	Foot abduction/adduction (deg)	Oscillation amplitudes (Max-Min)

		JOINT OSCILLATION VELOCITY	
A	65	Whole limb oscillation velocity (deg/s)	Minimum angle velocity
A	66	Hip joint angle oscillation velocity (deg/s)	Minimum angle velocity
A	67	Knee joint angle oscillation velocity (deg/s)	Minimum angle velocity
A	68	Ankle joint angle oscillation velocity (deg/s)	Minimum angle velocity
A	69	Whole limb oscillation velocity (deg/s)	Maximum angle velocity
A	70	Hip joint angle oscillation velocity (deg/s)	Maximum angle velocity
A	71	Knee joint angle oscillation velocity (deg/s)	Maximum angle velocity
A	72	Ankle joint angle oscillation velocity (deg/s)	Maximum angle velocity
A	73	Whole limb oscillation velocity (deg/s)	Amplitude of angle velocity (Max - Min)
A	74	Hip joint angle oscillation velocity (deg/s)	Amplitude of angle velocity (Max - Min)
A	75	Knee joint angle oscillation velocity (deg/s)	Amplitude of angle velocity (Max - Min)
A	76	Ankle joint angle oscillation velocity (deg/s)	Amplitude of angle velocity (Max - Min)

		PC ANALYSIS
A	77	Degree of linear coupling between joint oscillations

		FFT DECOMPOSITION	
A	78	Temporal coupling between crest and thigh oscillation	proximal
A	79	Temporal coupling between thigh and leg oscillation	
A	80	Temporal couling between leg and foot oscillation	distal

		CROSS CORRELATION BETWEEN SEGMENTS	
A	81	Correlation between crest and thigh oscillation	proximal
A	82	Correlation between thigh and leg oscillation	
A	83	Correlation between leg and foot oscillation	distal
A	84	Correlation between hip and knee oscillation	proximal
A	85	Correlation between knee and ankle oscillation	
A	86	Correlation between ankle and foot oscillation	distal

		RELATIVE COUPLING BETWEEN SEGMENT	
A	87	Duration between maximal backward positions of crest and thigh (% of gait cycle duration)	proximal
A	88	Duration between maximal forward positions of crest and thigh (% of gait cycle duration)	
A	89	Duration between backward positions of thigh and leg (% of gait cycle duration)	
A	90	Duration between forward positions of the thigh and leg (% of gait cycle duration)	
A	91	Duration between backward positions of leg and foot (% of gait cycle duration)	
A	92	Duration between forward positions of leg and foot (% of gait cycle duration)	distal

		REPRODUCIBILITY OF ENDPOINT TRAJECTORY
A	93	Consistency of limb endpoint trajectory over 10 successive cycle in X plane
A	94	Consistency of limb endpoint trajectory over 10 successive cycle in Y plane
A	95	Consistency of limb endpoint trajectory over 10 successive cycle in Z plane
A	96	Consistency of limb endpoint trajectory over 10 successive cycle in XY planes
A	97	Consistency of limb endpoint trajectory over 10 successive cycle in XYZ planes

		COMPARISON TO REFERENCE JOINT ANGLES
A	98	Correlation between limb oscillation and reference limb oscillation
A	99	Correlation between hip oscillation reference hip oscillation
A	100	Correlation between knee oscillation reference knee oscillation
A	101	Correlation between ankle oscillation reference ankle oscillation
A	102	Correlation between foot oscillation reference foot oscillation
