# Supplementary material for: Neurotransmitter phenotype switching by spinal excitatory interneurons regulates locomotor recovery after spinal cord injury
Source: Nat Neurosci. 2022 May 6;25(5):617–29. doi: 10.1038/s41593-022-01067-9 (PMC9076533; doi:10.1038/s41593-022-01067-9)
Supplement: Supplementary file 2 — Reporting Summary [file 41593_2022_1067_MOESM2_ESM.pdf]

nature portfolio

Corresponding author(s): Aya Takeoka

Last updated by author(s): Mar 23, 2022

Reporting Summary

Nature Portfolio wishes to improve the reproducibility of the work that we publish. This form provides structure for consistency and transparency in reporting. For further information on Nature Portfolio policies, see our Editorial Policies and the Editorial Policy Checklist.

Statistics

For all statistical analyses, confirm that the following items are present in the figure legend, table legend, main text, or Methods section.

n/a	Confirmed	
☐	☒	The exact sample size (*n*) for each experimental group/condition, given as a discrete number and unit of measurement
☐	☒	A statement on whether measurements were taken from distinct samples or whether the same sample was measured repeatedly
☐	☒	The statistical test(s) used AND whether they are one- or two-sided<br>*Only common tests should be described solely by name; describe more complex techniques in the Methods section.*
☐	☒	A description of all covariates tested
☐	☒	A description of any assumptions or corrections, such as tests of normality and adjustment for multiple comparisons
☐	☒	A full description of the statistical parameters including central tendency (e.g. means) or other basic estimates (e.g. regression coefficient) AND variation (e.g. standard deviation) or associated estimates of uncertainty (e.g. confidence intervals)
☐	☒	For null hypothesis testing, the test statistic (e.g. *F*, *t*, *r*) with confidence intervals, effect sizes, degrees of freedom and *P* value noted<br>*Give P values as exact values whenever suitable.*
☒	☐	For Bayesian analysis, information on the choice of priors and Markov chain Monte Carlo settings
☒	☐	For hierarchical and complex designs, identification of the appropriate level for tests and full reporting of outcomes
☒	☐	Estimates of effect sizes (e.g. Cohen's *d*, Pearson's *r*), indicating how they were calculated

Our web collection on statistics for biologists contains articles on many of the points above.

Software and code

Policy information about availability of computer code

Data collection	VICON Nexus 2.16 for locomotor kinematic data
Data analysis	All statistical analyses were performed and plotted using R (3.5), MATLAB (2018a) , GraphPad PRISM (v8.0), imaris (9.2.0), imageJ 2.1.0

For manuscripts utilizing custom algorithms or software that are central to the research but not yet described in published literature, software must be made available to editors and reviewers. We strongly encourage code deposition in a community repository (e.g. GitHub). See the Nature Portfolio guidelines for submitting code & software for further information.

Data

Policy information about availability of data

All manuscripts must include a data availability statement. This statement should provide the following information, where applicable:

- Accession codes, unique identifiers, or web links for publicly available datasets
- A description of any restrictions on data availability
- For clinical datasets or third party data, please ensure that the statement adheres to our policy

The data are available from the corresponding author on reasonable request.

Field-specific reporting

Please select the one below that is the best fit for your research. If you are not sure, read the appropriate sections before making your selection.

☒ Life sciences ☐ Behavioural & social sciences ☐ Ecological, evolutionary & environmental sciences

For a reference copy of the document with all sections, see nature.com/documents/nr-reporting-summary-flat.pdf

Life sciences study design

All studies must disclose on these points even when the disclosure is negative.

Sample size	No statistical methods were used to predetermine sample sizes. However, all behavioral and anatomical experiments were repeated using multiple animals, and sample sizes were similar to previous studies (i.e.,Takeoka and Arber 2019, Takeoka et al., 2014).
Data exclusions	Data exclusion/inclusion criteria are clearly described in the method section. Data were excluded from the study when 1) spinal cord injured mice showed muscle atrophy, 2) viral infection rate was low to modify gene expression, 3) suboptimal cell ablation upon diphtheria toxin administration, and 4) poor tissue sample preparation that could not provide any meaningful data for the analysis.
Replication	All attempts to replicate data were successful. Behavioral and anatomical results were replicated in multiple mice.
Randomization	Except for random sub-sampling of behavioral and anatomical data, randomization was not used during analysis. Animals (within genotype pools) were randomly assigned to experimental groups.
Blinding	Identical confocal imaging parameters were used for collecting anatomical data. Blinding was not used for anatomical and kinematic analyses, but automated analyses used were not subject to experimenter bias.

Reporting for specific materials, systems and methods

We require information from authors about some types of materials, experimental systems and methods used in many studies. Here, indicate whether each material, system or method listed is relevant to your study. If you are not sure if a list item applies to your research, read the appropriate section before selecting a response.

Materials & experimental systems

n/a	Involved in the study	
☐	☒	Antibodies
☒	☐	Eukaryotic cell lines
☒	☐	Palaeontology and archaeology
☐	☒	Animals and other organisms
☒	☐	Human research participants
☒	☐	Clinical data
☒	☐	Dual use research of concern

Methods

n/a	Involved in the study	
☒	☐	ChIP-seq
☒	☐	Flow cytometry
☒	☐	MRI-based neuroimaging

Antibodies

Antibodies used	chicken anti-beta-galactosidase (1:5000, Abcam AB9361), chicken anti-GFP (1:500, Molecular Probe A10262), goat anti-ChAT (1:1000, Chemicon AB144P), guinea pig anti-gephyrin (1:1000, SySy 147-318), guinea pig anti-vGlut1 (1:20000, Chemicon AB5905), mouse anti-glycine receptor alpha 1 (GlyRα1) (1:2000, SySy 146-111), mouse anti-NeuN (1:1000, Millipore MAB377), mouse anti-vesicular GABA Transporter (vGAT) (1:500, SySy 131-011), mouse anti-vesicular Glutamate Transporter 2 (vGlut2) (1:500, Merck Millipore MAB5504), rabbit anti-GABA (1:4000, Sigma A2052), rabbit anti-GFP (1:5000, Thermo Fisher Scientific A11122), rabbit anti-vesicular GABA Transporter (vGAT) (1:2000, Merck Millipore AB5062P), rabbit anti-vesicular Glutamate Transporter 2 (vGlut2) (1:500, SySy 135-403). Fluorophore-coupled secondary antibodies were obtained from Jackson and used at 1:1000: Alexa Fluor 488 donkey anti-chicken (703-545-155), Alexa Fluor 488 donkey anti-guinea pig (706-545-148), Alexa Fluor 488 donkey anti-mouse (715-545-150), Cy3 Donkey Anti-Goat (705-165-147), Cy3 Donkey Anti-Guinea Pig (706-165-148), Cy3 Donkey Anti-Mouse (715-165-150), Cy3 Donkey Anti-Rabbit (711-165-152), Alexa Fluor 647 donkey anti-goat (705-605-147), Alexa Fluor 647 donkey anti-mouse (715-605-150), Alexa Fluor 647 donkey anti-rabbit (711-605-152).
Validation	Antibodies were validated by examining vendor statistics and controls when available (Molecular Probe (GFP) 10262: Vendor validation by Western Blot analysis; Chemicon (ChAT) AB144P: Quality Assurance by Western Blot analysis; SySy (Gephyrin) 131-01: Vendor validation by Western Blot images; Merck Millipore (vGlut2) MAB5504: Quality Assurance by Western Blot analysis; Thermo Fisher Scientific (GFP) A11122: Vendor validation by Western Blot analysis; SySy (vGlut2) 135-403: Vendor validation by Western Blot images). If antibody was not listed, the way of validation was not explicitly mentioned however it was stated they were validated for immunohistochemistry. Furthermore, quality of images was carefully examined for background staining and nonspecific labeling. Thorough literature research has been conducted before usage of new antibodies. All antibodies used in this study have been used in previous publications: Anti-[illegible]galactosidase, Abcam AB9361: Tröster P et al., (2018), Mol Neurosci 11:19; Anti-GFP, Molecular Probe

A10262: Takeoka and Arber, (2019), Cell Rep. 27(1), 71-85; Anti-ChAT, Chemicon AB144P: Takeoka A et al., (2010), Experimental neurology 222(1), 59-69; Anti-Gephyrin, SySy 147-318: Pan H et al., (2019), Neuron 103(6):1135-1149; Anti-vGlut1, Chemicon AB5905: Sakurai K et al., (2013), Cell rep. 5(1), 87-89; Anti-GlyRα1, SySy 146-111:Sinha R et al., (2021) Curr. Biol. 31(19), 4314-4326; Anti-NeuN, Millipore MAB377: Ding Y et al., (2014) Plos one 9(7), e101918; Anti-vGAT, SySy 131-01: Pan H et al., (2019), Neuron 103(6):1135-1149; Anti-vGlut2, Merck Millipore MAB5504: Duvick L et al., (2010), Neuron 67(6), 929-935; Anti-GABA, Sigma A2052: Dougherty K J et al., (2009) Neuroscience 163(3), 909-919; Anti-GFP, Thermo Fisher Scientific A11122: Choi S et al., (2020) Nature 587(7833), 258-263; Anti-vGAT, Merck Millipore AB5062P: Mclntire S et al., (1997) Nature 389(6653), 870-876; Anti-vGlut2, SySy 135-403: Mullen P et al., (2022), eNeuro 9(1)).

Animals and other organisms

Policy information about studies involving animals; ARRIVE guidelines recommended for reporting animal research

Laboratory animals	Wild-type (C57Bl6), PVcre (JAX stock #008069), AdvilliniDTR (Stantcheva et al., 2016), vGlut2cre (JAX stock #028863), vGATcre (JAX stock #016962), Tlx3cre (041158-UCD), Shox2cre (Dougherty et al., 2013), Sim1cre (Zhang et al., 2008), Rosa26LSL-tdTomato (JAX stock #007908), and TauLSL-nlsLacZ-SynGFP-INLA (Tripodi et al., 2014) mouse strains were maintained on a mixed genetic background (129/C57Bl6). Adult mice of both sexes, aged 2–6 months, were used for all experiments. Mice were group housed, and all mice were kept under a 12 hour light/dark cycle in a facility where temperature was kept at 22 ± 2 degrees Celsius and ~50% humidity.
Wild animals	No wild animals were used in this study.
Field-collected samples	No field-collected samples were used in this study.
Ethics oversight	All animal procedures were conducted in accordance with Belgian regulations and approved the institutional ethical committee of KU Leuven.

Note that full information on the approval of the study protocol must also be provided in the manuscript.
